# Black soldier fly (*Hermetia illucens*) larva meal as a sustainable protein enhances growth, health and flesh quality of *Scyllaparamamosain*

**DOI:** 10.1016/j.aninu.2025.11.016

**Published:** 2026-06-08

**Authors:** Tiantian Xu, Yuhang Yang, Xiaoyi Zhao, Shichao Xie, Yinqiu Tian, Tingting Zhu, Min Jin, Qicun Zhou

**Affiliations:** a Laboratory of Fish and Shellfish Nutrition, School of Marine Sciences, Ningbo University, Ningbo 315211, Zhejiang, China; b Key Laboratory of Aquacultural Biotechnology Ministry of Education, Ningbo University, Ningbo 315211, Zhejiang, China; c Key Laboratory of Green Mariculture (Co-Construction by Ministry and Province), Ministry of Agriculture and Rural, Ningbo University, Ningbo 315211, Zhejiang, China; d Collaborative Innovation Center for Zhejiang Marine High-Efficiency and Healthy Aquaculture, Ningbo 315211, Zhejiang, China

**Keywords:** *Scylla paramamosain*, Black soldier fly larvae meal, Health, Nutrient composition, Flesh quality

## Abstract

The optimal level of fish meal (FM) replacement with black soldier fly (*Hermetia illucens*) larva meal (BSFM) in mud crab (*Scylla paramamosain*), particularly in terms of health, texture, and flavor quality, remains unclear. This study aimed to systematically evaluate the effects of substituting FM with BSFM on the growth performance, health, and flesh quality of mud crabs. Six isonitrogenous and isolipidic diets were formulated replacing FM with BSFM at 0, 10%, 20%, 30%, 40%, and 60%. A total of 192 crabs with an initial weight of 22.46 ± 0.04 g were randomly assigned to four replicates per treatment, with eight crabs per replicate, and cultured for 8 weeks. The findings indicated that BSFM as a FM substitute posed no risk of heavy metal or pesticide contamination. Substituting up to 30% of FM with BSFM did not adversely affect the growth or nutritional value of mud crabs compared to the substitution of FM with 0 BSFM (BSFM0) group (*P >* 0.05). Notably, moderate inclusion of BSFM increased the fatty acid content in the hepatopancreas, enhanced the hardness, springiness, chewiness, and gumminess, and increased the collagen content in the muscles of mud crabs compared to the BSFM0 group (*P* < 0.05). However, a BSFM replacement level of over 40% reduced the length and width of intestinal folds, decreased the thickness of intestinal muscularis, and caused detachment of intestinal villi, causing intestinal damage in mud crabs compared to the BSFM0 group (*P* < 0.05). High levels of BSFM substitution led to a reduction in amino acids, free amino acids (umami free amino acids), flavor-related nucleotides (adenosine monophosphate and hypoxanthine), and sensory scores (umami, sweetness, and saltiness values), causing a reduction in the nutritional quality and flavor of the crab flesh compared to the BSFM0 group (*P* < 0.05). Overall, this study suggests that replacing 30% of FM with BSFM can promote growth, and improves health and flesh quality in mud crabs.

## 1 Introduction

Aquaculture plays an essential role in meeting the increasing worldwide demand for animal protein. Aquatic animals, including finfish, crustaceans, and molluscs, are rich in protein and essential micronutrients, contributing significantly to the diets of over 4.5 billion people worldwide (Mohanty et al., 2019). The aquaculture sector has expanded substantially in recent years, propelled by rising worldwide demand for animal protein and innovations in farming practices. Production reached 122.6 million tons in 2020, among them, Asia, particularly China, accounted for more than 90% of the global aquaculture output and led this growth (FAO, 2022). Feed constitutes the largest operational cost (60%–70%) in aquaculture production. Fish meal (FM) has traditionally been considered as a high-quality protein source due to its rich amino acid and fatty acid profiles. However, the heavy reliance on marine capture for FM has led to the overexploitation of marine resources. Consequently, the aquaculture industry is increasingly seeking sustainable protein sources to reduce FM usage, driven by its scarcity and high cost (Naylor et al., 2021; Kim et al., 2019; Panini et al., 2017). Researchers are actively exploring FM alternatives that meet sustainability standards, recognizing that FM can be partially or fully replaced in aquaculture feeds (Zarantoniello et al., 2023).

Black soldier fly (*Hermetia illucens*) larval meal (BSFM) is emerging as a viable alternative feed ingredient for both terrestrial and aquatic animal diets (Albayati et al., 2025; Bruni et al., 2020b; Lanes et al., 2021; Jiang et al., 2022; Mikolajczak et al., 2022; Villanueva-Gutiérrez et al., 2022). The BSFM production offers significant environmental benefits, such as the efficient conversion of organic waste into high-quality biomass, diminished land and water use, and decreased greenhouse gas emissions, thereby supporting a circular economy (Anedda et al., 2023; Diener et al., 2009). From a nutritional standpoint, fresh black soldier fly (BSF) larvae have a dry matter content ranging from 35% to 45%. The crude protein content of full-fat BSFM is less than 40%, while partially defatted BSFM contains more than 56% crude protein (Makkar et al., 2014; Villanueva-Gutiérrez et al., 2022). The BSFM boasts a well-balanced profile of essential amino acids, along with high levels of vitamins and minerals. Compared to plant proteins, BSFM is particularly rich in methionine, lysine, and leucine, and it is free of anti-nutritional factors (ANFs). Furthermore, BSFM comprises bioactive components, including chitin, lauric acid, and antimicrobial peptides, which may contribute to enhancing intestinal immunity and anti-inflammatory responses in animals (Belghit et al., 2019; Gaudioso et al., 2021; Spranghers et al., 2017). However, BSF larvae have the potential to accumulate harmful substances such as heavy metals and pesticides from their rearing substrates. These contaminants can subsequently enter the feed supply chain, thereby posing potential risks (Charlton et al., 2015). Among these, organochlorine pesticides (OCPs) can persist and bioaccumulate through the food chain, posing serious health threats (Li et al., 2020). Previous research indicated that BSF larvae are capable of excreting various contaminants, although Cd and Pb tend to accumulate in their bodies (Alagappan et al., 2022; Purschke et al., 2017). The rearing substrate plays a fundamental role in determining the nutritional potential and safety of black soldier fly larvae (Ganesan et al., 2024; Navajas-Porras et al., 2024; Sangiacomo et al., 2025). Therefore, using clean substrates and implementing stringent management practices are crucial for producing safe, healthy BSF.

Critically, the dietary composition, including the inclusion of alternative ingredients like BSFM, is a major external factor determining flesh quality, a composite indicator encompassing nutritional content (such as protein, lipids, amino acids, and fatty acids), flavor profile (taste and aroma), texture characteristics (including texture and liquid holding capacity), and safety indicators (such as heavy metals and contaminants) (Huang et al., 2024; Picard et al., 2012). Although FM replacement with BSFM has been studied in several finfish species, such as pikeperch (*Sander lucioperca*), rice field eel (*Monopterus albus*), and red sea bream (*Pagrus major*) (Hu et al., 2020; Stejskal et al., 2023; Takakuwa et al., 2022), and has shown promising results in maintaining or even improving growth and flesh quality (Bruni et al., 2020a; Hu et al., 2023; Pulido et al., 2022), research in crustaceans remains scarce. This is particularly true for the mud crab (*Scylla paramamosain*), a high-value species prized for its distinctive texture and delicate flavor, which commands premium prices in international markets (Tang et al., 2024). Therefore, the effect of substituting FM with BSFM on crab flesh quality represents an important and yet unexplored research gap.

Mud crabs are highly valued for their flavor, nutritional content, and economic importance in coastal aquaculture, particularly in regions such as the Indian Ocean and southeastern China (Zheng et al., 2020). The flesh quality of mud crabs, including their texture, flavor, and nutritional composition, is a key determinant of their market value. Factors such as amino acid profile, lipid content, and mineral levels provide a vital function in determining both the sensory attributes and health benefits of the meat. Despite the growing interest in BSFM as a sustainable protein source, critical knowledge gaps hinder its evidence-based application in crab aquaculture. Firstly, a systematic evaluation of the impact of BSFM on the overall flesh quality of crabs, encompassing texture, flavor compounds, and nutritional profile, remains lacking. Secondly, the safety aspect of BSFM-derived contaminants accumulating in crab tissues remains unverified for this species. Finally, the optimal inclusion level of BSFM that maximizes benefits without inducing negative effects on intestinal health or overall quality is unknown. This study investigated the effects of substituting FM with BSFM in mud crab diets on the growth, safety, intestinal health, and multiple aspects of flesh quality of mud crabs. The study aimed to provide insights for exploring sustainable use of BSF-based ingredients in aquaculture while supporting the production of high-quality aquatic products.

## 2 Materials and methods

### 2.1 Animal ethics statement

The study was approved by the Ningbo University Animal Research and Ethics Committee (No. SYXK20190005), and conducted in strict adherence to Standard Operating Procedures (SOPs) outlined in Ningbo University's guidelines for the ethical treatment of experimental animals. Additionally, the experimental protocols received formal approval from Ningbo University's Institutional Animal Care and Use Committee, ensuring compliance with ethical standards.

### 2.2 BSFM preparation

The BSFM was purchased from Bioforte Biotechnology Co., Ltd. (Shenzhen, Guangdong, China). The BSF larvae were reared on a substrate composed of residual kitchen waste and larval frass (a mixture of excreta and secretions). The black soldier fly oil (BSFO) was derived from the same batch of prepupae used to produce the BSFM, ensuring consistency of source. Briefly, the larvae meal was mixed with petroleum ether at a ratio of 1:12 (w/w). The extraction was conducted at 60 °C for 180 min, following a pre-drying step of 30 min. To ensure complete removal of the petroleum ether solvent, the extracted oil was placed in an oven (DHG-9240A, Shanghai Yiheng Scientific Instrument Co., Ltd., Shanghai, China) at 50 °C with the lid open until a constant weight was achieved. The final oil was stored at −20 °C for subsequent use. The fatty acid profiles of the BSFO and FO are shown in Table 1.

**Table 1:** Fatty acids composition of the FO and BSFO (% of total fatty acids).

Fatty acids	FO	BSFO
C12:0	0.06	13.12
C14:0	4.23	4.81
C16:0	19.52	23.00
C18:0	5.57	4.09
C20:0	0.49	0.11
ΣSFA	29.81	45.12
C16:1n	4.91	2.50
C18:1n-9	28.97	37.92
C20:1n-9	1.25	0.20
C22:1n-11	0.21	0.00
ΣMUFA	35.35	40.62
C18:2n-6	9.21	9.69
C18:3n-6	0.10	0.05
C20:2n-6	0.22	0.12
C20:4n-6	1.21	0.54
Σn-6 PUFA	10.73	10.39
C18:3n-3	2.91	2.95
C18:4n-3	0.87	0.10
C20:4n-3	0.43	0.10
C20:5n-3	5.05	0.62
C22:5n-3	1.02	0.03
C22:6n-3	13.84	0.05
Σn-3 PUFA	24.11	3.86

FO = fish oil; BSFO = black soldier fly larvae oil; SFA = saturated fatty acids; MUFA = mono-unsaturated fatty acids; PUFA = polyunsaturated fatty acids.

### 2.3 Diet preparation

This experiment involved the formulation of six isonitrogenous diets (46% crude protein) and isolipidic diets (10% crude lipid) were developed to suit the unique dietary needs of mud crabs, as previously advised (Luo et al., 2021). These diets included five experimental diets with low-fat BSFM substituting 10%, 20%, 30%, 40%, and 60% of FM, as well as a control diet (BSFM0) with 30% FM (Table 2). As the level of FM replacement with BSFM increased, the water stability of the experimental diet in water (at 60 and 120 min) showed a significant decreasing trend. However, the water stability at 120 min remained above 84% across all treatments (Table 3). Table 4 details the amino acid contents of the BSFM, FM, and experimental diets. The fish oil used was commercially available anchovy oil purchased from Ningbo Tech-Bank Corp., Ningbo, Zhejiang, China. All the materials were processed into a fine powder with particles smaller than 177 μm. The components were accurately weighed in accordance with the experimental formulation. Firstly, trace elements, such as mineral and vitamin premixes, were carefully blended through a stepwise scale-up process, followed by the addition of lipids and distilled water (approximately 35% w/w). The mixture was then combined with FM, krill meal, and the remaining ingredients and further homogenized using a Hobart mixer (HL600, Hobart Co., Troy, NY, USA). Pellets with homogeneous diameters of 1.5 and 2.5 mm were extruded using a twin-screw extruder (F-26, South China University of Technology, Guangzhou, Guangdong, China). The prepared diets were dried in an oven at 45 °C until they reached a moisture content of approximately 10%. Subsequently, they were vacuum-packed and stored at −20 °C until use.

**Table 2:** Ingredients and proximate composition of experimental diets (based on wet basis).

Items	Replacement of FM with BSFM, %					
	0	10	20	30	40	60
**Ingredients, g/100 g**						
Peru fish meal^1^	30.00	27.00	24.00	21.00	18.00	12.00
Krill meal^1^	3.00	3.00	3.00	3.00	3.00	3.00
BSFM^2^	0.00	4.08	8.18	12.25	16.34	24.52
Soybean meal^1^	18.00	18.00	18.00	18.00	18.00	18.00
Soybean protein concentrate^1^	15.00	15.00	15.00	15.00	15.00	15.00
Wheat flour^1^	16.79	16.79	16.79	16.79	16.79	16.79
Lysine^3^	0.00	0.04	0.07	0.11	0.15	0.22
Methionine^3^	0.00	0.01	0.03	0.05	0.06	0.09
Fish oil^1^	0.00	0.35	0.69	1.04	1.39	2.08
BSF larvae oil^2^	3.01	2.51	2.00	1.51	1.00	0.00
Cholesterol^3^	0.50	0.50	0.50	0.50	0.50	0.50
Soybean lecithin^1^	2.00	2.00	2.00	2.00	2.00	2.00
Vitamin premix^4^	0.50	0.50	0.50	0.50	0.50	0.50
Mineral premix^4^	1.00	1.00	1.00	1.00	1.00	1.00
Ca(H_2_PO_4_)_2_^3^	2.00	2.00	2.00	2.00	2.00	2.00
Choline chloride^1^	0.30	0.30	0.30	0.30	0.30	0.30
Sodium alginate^1^	2.00	2.00	2.00	2.00	2.00	2.00
Cellulose^1^	5.90	4.92	3.93	2.95	1.97	0.00
Total	100.00	100.00	100.00	100.00	100.00	100.00
**Proximate composition**^5^						
Dry matter, %	93.85	94.09	94.34	94.71	94.68	94.76
Crude protein, %	43.29	43.34	43.75	43.74	43.76	43.61
Crude lipid, %	10.88	10.19	10.33	10.39	10.48	10.78
Organic matter, %	90.30	90.19	89.94	89.86	89.83	89.76
Crude fiber, %	6.66	5.79	4.91	4.03	3.14	1.36
Gross energy, kJ/g	17.55	17.55	17.55	17.55	17.55	17.55
Fiber/gross energy, g/MJ	4.05	3.50	2.96	2.43	1.89	0.81

FM = fish meal; BSFM = black soldier fly larvae meal; BSF = black soldier fly.

1 Peru fish meal (crude protein:65.45%, crude lipid:10.71%, based on wet basis) from Ningbo Tech-Bank Corp., Ningbo, Zhejiang, China.

2 Black soldier fly larvae meal (crude protein:49.18%, crude lipid:12.04%, based on wet basis) from Bioforte Biotechnology Co., Ltd., Shenzhen, Guangdong, China.

3 Macklin Biochemical Co., Ltd., Shanghai, China.

4 Mineral premix and vitamin premix were purchased from Ningbo Tech-Bank Co., Ltd. (Ningbo, Zhejiang, China). Mineral premix (g/kg): FeC_6_H_5_O_7_, 4.57; ZnSO_4_·7H_2_O, 9.43; MnSO_4_·H_2_O (99%), 4.14; CuSO_4_·5H_2_O (99%), 6.61; MgSO_4_·7H_2_O (99%), 238.97; KH_2_PO_4_, 233.2; NaH_2_PO_4_, 137.03; C_6_H_10_CaO_6_·5H_2_O (98%), 34.09; CoCl_2_·6H_2_O (99%), 1.36. Vitamin premix supplied the diet with (g/kg): retinyl acetate, 1.2121; cholecalciferol, 1.2000; all-rac-α-tocopherol, 20.0000; menadione, 9.0909; thiamine, 10.8696; riboflavin, 7.5000; ascorbic acid, 30.0000; pyridoxine hydrochloride, 12.1212; cyanocobalamin, 2.0000; folic acid, 40.0000; biotin, 12.5000; nicotinic acid, 40.4040; D–Ca pantothenate, 16.1290; inositol, 204.0816; cellulose, 592.8915.

5 Dry matter, crude lipid, gross energy, crude fiber, and organic matter were analyzed.

**Table 3:** Water stability (%) of the experimental diets (based on wet basis).

Time, min	Replacement of FM with BSFM, %						SEM	*P-*value		
	0	10	20	30	40	60		ANOVA	Linear	Quadratic
60	93.36^a^	93.08^ab^	93.24^a^	93.21^a^	92.38^bc^	92.22^c^	0.120	0.001	<0.001	0.038
120	86.67^a^	85.94^ab^	85.15^abc^	85.08^abc^	84.32^bc^	83.73^c^	0.271	0.002	<0.001	0.823

FM = fish meal; BSFM = black soldier fly larvae meal; SEM = standard error of the mean.

Within a row, means without a common superscript letter differ at *P* < 0.05, *n* = 4.

**Table 4:** Amino acid compositions of BSFM, FM, and experimental diets (based on wet basis).

Amino acids	Ingredients		Replacement of FM with BSFM, %					
	BSFM	FM	0	10	20	30	40	60
**EAA, g/100 g**								
Arginine	2.24	3.73	2.51	2.47	2.44	2.43	2.40	2.34
Histidine	1.35	2.13	1.10	1.08	1.08	1.07	1.06	1.03
Isoleucine	2.07	2.40	1.72	1.68	1.64	1.64	1.62	1.58
Leucine	3.21	4.80	3.12	3.06	3.03	3.01	2.97	2.90
Lysine	2.81	4.99	2.53	2.52	2.63	2.66	2.53	2.57
Methionine	0.36	1.71	0.71	0.73	0.71	0.69	0.69	0.69
Phenylalanine	1.94	2.54	1.87	1.85	1.86	1.87	1.81	1.82
Threonine	1.77	2.75	1.62	1.59	1.59	1.59	1.55	1.54
Valine	2.51	2.84	1.90	1.88	1.92	1.93	1.86	1.90
ΣEAA	18.26	27.87	17.07	16.87	16.91	16.91	16.48	16.38
**NEAA**								
Alanine, g/100 g	2.72	4.06	2.12	2.08	2.08	2.06	2.03	2.00
Aspartic acid, g/100 g	4.14	5.72	3.91	3.87	3.89	3.92	3.80	3.81
Cysteine, g/100 g	0.28	0.49	0.37	0.37	0.36	0.37	0.38	0.37
Glutamic acid, g/100 g	5.48	8.44	6.94	6.84	6.81	6.87	6.67	6.64
Glycine, g/100 g	2.25	3.75	2.05	2.00	1.99	1.97	1.94	1.88
Proline, g/100 g	2.18	5.52	1.97	1.90	1.93	1.98	1.88	1.97
Serine, g/100 g	1.81	2.55	1.78	1.77	1.79	1.80	1.74	1.72
Tyrosine, g/100 g	2.43	1.96	1.90	1.88	1.92	1.93	1.86	1.81
ΣNEAA, g/100 g	21.30	32.50	21.04	20.71	20.77	20.89	20.29	20.19
ΣEAA/ΣNEAA	0.84	0.80	0.76	0.76	0.76	0.77	0.77	0.77

FM = fish meal; BSFM = black soldier fly larvae meal; EAA = essential amino acid; NEAA = non-essential amino acid.

### 2.4 Feeding trial and experimental conditions

Healthy juvenile mud crabs were obtained from a crab farm in Sanmen, Taizhou, Zhejiang, China. This experiment was conducted in the crab facility of the indoor recirculating aquaculture system (RAS) at Ningbo University (Ningbo, Zhejiang, China). Individual crab apartment boxes (0.45 m × 0.30 m × 0.20 m) were used for precise monitoring and management in this study. Mud crabs were acclimatized for 14 d with a commercial feed (Ningbo Tech-Bank Corp., Ningbo, Zhejiang, China) containing 45% protein and 8% lipid before the experiment. Each crab (weighing 22.46 ± 0.04 g) was randomly assigned to one of 192 crab apartment boxes. Four 8-crab replicates were assigned to each of the six dietary treatments, and the crabs were cultured for 8 weeks. The feeding protocol for experimental crabs according to Yang et al. (2025) and Luo et al. (2020). They were fed to satisfaction once daily at 17:30. Dead crabs were weighed and recorded every morning at 08:30 to track mortality. The RAS has a water capacity of 6 tons, with 10% of the total volume automatically replaced daily with fresh seawater. This system achieves a recirculation rate of 8 times daily, ensuring a high water exchange rate within the tanks. The culture water was sourced from natural seawater, which was sterilized and filtered through a dedicated water preparation system (including sediment filters, carbon filters, and UV sterilization) before being introduced into the system. The experiment monitored these environmental conditions: temperature 26.3 to 27.5 °C, salinity 22.4‰ to 23.8‰, dissolved oxygen 6.5 to 7.0 mg/L, and ammonia nitrogen below 0.05 mg/L. Salinity, temperature, ammonia nitrogen, and dissolved oxygen were measured with a YSI Proplus instrument (YSI Incorporated, Yellow Springs, OH, USA). The photoperiod was 10L:14D (photo phase from 07:00-17:00), and the light intensity was 200 lx.

### 2.5 Sample collection

At the end of the experiment, surviving crabs were weighed and counted after feeding to assess growth performance. Three crabs per replicate were anesthetized on ice. Hemolymph was collected from each crab (3 crabs/replicate) using a 1-mL syringe (D10-1, Xinjin Shifeng Medical Apparatus in Instrument Co., Ltd., Chengdu, Sichuan, China) and transferred to a 1.5-mL centrifuge tube. The samples were kept at 4 °C overnight and then centrifuged the following day (4 °C, 587 × *g*, 10 min) using a centrifuge (3–30 KS, Sigma Laborzentrifugen GmbH, Osterode am Harz, Germany). The supernatant was stored at −80 °C for subsequent analysis. Hepatopancreas and intestine tissues (approximately 300 mg/crab, 3 crabs/replicate) were collected from the same three crabs mentioned above and immediately preserved in RNA-later solution in 1.5-mL tubes. The remaining portions of the tissues (hepatopancreas and muscle) from the same three crabs were collected into separate 2-mL tubes, rapidly frozen in liquid nitrogen, and stored at −80 °C. These samples were used for the analysis of conventional composition, amino acid profile, fatty acid profile, hydrolyzed and free amino acids, nucleotides, hydroxyproline, inorganic ions, and electronic-tongue analysis. Fresh intestinal and muscle tissues were collected from two different crabs per replicate. These tissues (2 crabs/replicate) were fixed in 4% paraformaldehyde for paraffin section preparation. Fresh meat from the swimming foot of crabs (3 crabs/replicate) was collected for immediate analysis of texture profile, pH, and liquid holding capacity (LHC).

### 2.6 Sample analysis

#### 2.6.1 Proximate composition analysis

The proximate composition of experimental diets, as well as the hepatopancreas and muscle of mud crabs, was determined using AOAC (2006) techniques. In a nutshell, crude lipid content was estimated using an extraction analyzer (SX360, OPSIS AB, Furulund, Skåne, Sweden) based on the Soxhlet extraction technique (method 920.39). By a protein analyzer (FP-528, Leco Corporation, San Jose, CA, USA), the crude protein content was analyzed using Dumas combustion techniques (method 968.06). The samples were dried to a constant weight at 105 °C to estimate the moisture content (method 934.01), and the ash concentration was determined using an 8-h muffle furnace (TM-2010, Beijing Innochem Science & Technology Co., Ltd., Beijing, China) operated at 550 °C (method 942.05). Crude fiber (method 962.09) was determined through sequential acid/alkaline digestion, filtration, degreasing, and gravimetric analysis of ash-free residues. Gross energy was analyzed via bomb calorimetry (ZDHW-ZC8000, Hebi Zhongchuang Instrument Co., Ltd., Hebi, Henan, China) (Rodríguez-Añón and Proupin-Castineiras, 2005). Organic matter content was calculated as dry matter minus ash. All components (dry matter, crude lipid, gross energy, crude fiber, and organic matter) were quantified gravimetrically.

#### 2.6.2 Water stability

The water stability of the experimental diets was determined according to the method described by Eide et al. (2024). Briefly, 10 g of feed was placed in a prepared cylindrical mesh sieve, which was then immersed in a water container with a depth of 5.5 cm at a temperature of 25 ± 2 °C for 60 and 120 min. During this period, the sieve was slowly lifted to the water surface and then slowly submerged back into the water three times to help release the feed from the bottom of the sieve. After immersion, the sieve was removed, tilted to drain excess water, and the remaining feed was dried in an oven (DHG-9240A) at 105 °C until a constant weight was achieved. Simultaneously, a separate sample of the same unimmerzed feed was dried under the same conditions to determine the initial moisture content. The water stability was calculated using the following formula:

$$\text{Water}\;\text{stability}\;\left(\%\right)=100\;–\;\left[m_{1}\times \left(1\;–\;x\right)\;–\;m_{2}\right]/\left[m_{1}\times \left(1\;–\;x\right)\right]\times 100\text{,}$$

where *m*_1_ is the initial 10 g feed weighed; *m*_2_ is the weight of the feed after soaking and drying; *x* is the moisture content of the feed.

#### 2.6.3 Safety inspections

All detected contaminants in BSFM, including hexachlorocyclohexane (HCH), dichlorodiphenyltrichloroethane (DDT), Pb, and Cd, satisfied the statutory limits (GB 13078–2017, China National Standard, 2017). Similarly, Pb and Cd concentrations in crab tissues met the maximum permissible levels, as referred to in (EC) No 1881/2006 (European Commission, 2006) and GB 2762-2022 (China National Standard, 2022). The detection methods for heavy metals refer to Hadji et al. (2016). Freeze-dried samples (0.5–1.0 g) were acid-digested with aqua regia (HCl/HNO_3_ = 1:3, v/v) at 80 °C until complete digestion of organic matter. The concentrations of Pb and Cd were determined using Inductively Coupled Plasma Mass Spectrometry (ICP-MS, NexION 300X, PerkinElmer Inc., Shelton, CT, USA). For quality assurance, certified reference material (GBW10023, fish tissue) was included in each batch of digestion and analysis. The average recovery rates for Pb and Cd were 95.2% and 102.4%, respectively. Method blanks were processed simultaneously to correct for potential background contamination. The limits of detection (LOD) were 0.4 μg/kg for Pb and Cd, and the limits of quantification (LOQ) were 1 μg/kg for Pb and Cd.

The detection methods for pesticide residues were conducted according to Qian et al. (2006). The analysis of HCH and DDT residues was performed by Agilent gas chromatography (7890B, Agilent Technologies Inc., Santa Clara, CA, USA) equipped with an electron capture detector. A mixed standard solution of HCH isomers (α-, β-, γ-, δ-HCH) and DDT metabolites (p,p'-DDE, p,p'-DDD, o,p'-DDT, p,p'-DDT) was used for calibration. Quality control included the analysis of procedural blanks and spiked samples with each batch. The average recoveries for HCH and DDT ranged from 88% to 105%. The LOD for HCH and DDT isomers was 0.20 mg/kg and 0.05 mg/kg, respectively.

#### 2.6.4 Amino acid analysis

The analysis of amino acid profiles in muscle and hepatopancreas was referenced by Yuan et al. (2020). Briefly, in an abbe bottle, lyophilized hepatopancreas and muscle samples were combined with 5 mL of 6 mol/L HCl solution. After that, the mix was broken down in a sand bath at 110 °C for 24 h. Following digestion, the samples were diluted in 0.02 mol/L HCl, filtered through a 0.22-μm aqueous phase filtration membrane, and put in the top sample tube. A high-speed amino acid analyzer (L8900, Hitachi High-Technologies Corporation, Tokyo, Japan) was used to measure the amino acid makeup. The sodium citrate buffer system was used, as shown in Table S2.

The free amino acid (FAA) composition was determined using the previously reported technique, with minor changes (Zhuang et al., 2016). One gram of samples was mixed with 15 mL of 5% trichloroacetic acid (TCA). It was then kept at 4 °C for 2 h after being ultrasonically extracted for 15 min. The supernatant was collected after spinning at 10,000 × *g* for 10 min at 4 °C. The TCA was then used to lower the pH to 2.2. Add 10 mL of water to the 5 mL residue and pass it through a 0.22-μm membrane. Without the lithium citrate buffer, FAA was examined in the same way that was already explained. High-performance liquid chromatography (HPLC) straight gradient recovery was shown in Table S3.

#### 2.6.5 Fatty acid analysis

The fatty acid content in the hepatopancreas and muscle samples of mud crab was measured using the technique reported by Xu et al. (2024). Briefly, the sample concentrator was dried in a tube with 1 mL tricosanoic acid (T6543, Sigma Chemical Co., St. Louis, MO, USA). Freeze-dried sample (100 mg) was mixed with 4 mL methanol-sulfuric acid solution in a glass tube. The mixture was then heated in water at 80 °C for 4 h. The mixture was then heated in water at 80 °C for 4 h. After cooling, 1 mL of hexane and 1 mL of distilled water were added, mixed thoroughly, and centrifuged at 587 × *g* for 1 min. The upper layer was filtered using a lipid-phase filter (SCAA-104, ANPEL Laboratory Technologies [Shanghai] Inc., Shanghai, China) and evaporated under a nitrogen stream. The residue was resuspended in 0.5 mL hexane and analyzed using gas chromatography-mass spectrometry (GC–MS; 7890B-5977A, Agilent Technologies Inc., Santa Clara, CA, USA).

#### 2.6.6 Health indicators analysis

##### 2.6.6.1 Hepatopancreas oxidative and antioxidant analysis

Hepatopancreas samples were processed as follows: samples were carefully weighed and homogenized in ice-cold saline (1:9, w/v) in an ice bath, followed by centrifugation (5810R, Eppendorf SE, Hamburg, Germany) at 1200 × *g* for 10 min at 4 °C. The supernatant was collected for enzyme analysis. The concentrations of catalase (CAT; A007-1-1), superoxide dismutase (T-SOD; A001-1-1), glutathione (GSH; A006-1-1), glutathione peroxidase (GSH-Px; A005-1-1), malondialdehyde (MDA; A003-1-1), protein carbonyl (PC; A087-1-1), and total protein (TP; A045-2-1) were determined using commercial detection kits (Nanjing Jiancheng Bioengineering Institute, Nanjing, Jiangsu, China). Absorbance was measured using a full-wavelength enzyme labelling instrument (Specramx M2, Molecular Devices, Sunnyvale, CA, USA).

##### 2.6.6.2 Histomorphology analysis

Fresh muscle and intestinal samples (2 crabs/replicate) were fixed in 4% paraformaldehyde and sent to Haoke Biotechnology Co., Ltd. (Hangzhou, Zhejiang, China) for processing. After being dried in graded ethanol, tissues were fixed in paraffin and cut into 4 μm pieces. After the sections were stained with H&E, they were examined with a Nikon Eclipse CI microscope (Nikon Instruments Inc., Tokyo, Japan). Image Pro Plus 6.0 software (Media Cybernetics Inc., Rockville, MA, USA) was used to calculate the size of the muscle morphology images, measure the cross-sectional area, fiber quantity, and fiber diameter of the muscle fibers, and then use these values to calculate the fiber density. Each tissue sample was measured 10 times.

##### 2.6.6.3 Gene expression analysis

Following Jin et al. (2024), RNA isolation, complementary DNA (cDNA) synthesis, and quantitative real-time PCR (qRT-PCR) were performed. The RNA was isolated from tissues using Trizol (Nanjing Vazyme Biotech Co., Ltd., Nanjing, Jiangsu, China), and quantified using a spectrophotometer (NanoDrop 2000; Thermo Fisher Scientific Inc., Waltham, MA, USA). Reverse transcription was performed using the HiScript II-RT Reagent Kit (Nanjing Vazyme Biotech Co., Ltd., Nanjing, Jiangsu, China). Select stable β-actin as the housekeeping gene. The qRT-PCR was performed using specific primers for target genes (Table S4) using a LightCycler 480II (Roche Diagnostics GmbH, Basel, Switzerland). The relative expression levels of target genes were determined by 2^−ΔΔCt^ method (Livak and Schmittgen, 2001).

#### 2.6.7 Flesh texture profile analysis (TPA)

Flesh TPA was conducted using a texture analyzer (TA.new Plus, Technology Co., Ltd., Shenzhen, China) equipped with a cylinder probe (TA/5). The procedure followed Song et al. (2022) with slight modifications. Samples were compressed twice using a flat-ended cylindrical probe with a deformation of 60%. The parameters measured included hardness, springiness, chewiness, gumminess, resilience, and cohesiveness. The trigger force was set to 5 g, with a holding time of 3 s, and pre-test, test, and post-test speeds of 1 mm/s.

#### 2.6.8 Liquid holding capacity, pH, and collagen content analysis

The method of Rørå et al. (2003) was adapted and slightly modified to determine LHC. The mathematical expressions:

$$\text{Liquid}\;\text{loss}=100\times \left(W_{2}\;–\;W_{1}\right)/M\text{;}$$

$$\text{Water}\;\text{loss}=100\times \left(W_{2}\;–\;W_{3}\right)/M\text{;}$$

$$\text{Lipid}\;\text{loss}=100\times \left(W_{3}\;–\;W_{1}\right)/M\text{;}$$

where *W*_*1*_ represents the weight of the filter paper after being dried at 80 °C. The muscle sample (*M* g) was wrapped in dry filter paper and placed into a 50 mL centrifuge tube. It was centrifuged at 500 × *g* for 10 min, after which the muscle tissue was discarded. *W*_*2*_ represents the weight of the filter paper at this time, and *W*_*3*_ represents the weight of the filter paper after it is dried at 80 °C. One gram of muscle tissue was weighed and transferred to a centrifuge tube. After adding 10 mL of distilled water, the sample was homogenized, and another 10 mL of distilled water was supplemented prior to a second homogenization. The pH of muscle samples was measured using a pH meter (PHS-25, INESA Scientific Instrument Co., Ltd., Shanghai, China). The hydroxyproline content, a marker for collagen, was measured using an assay kit (A030-3-1, Nanjing Jiancheng Bioengineering Institute, Nanjing, Jiangsu, China). The hydroxyproline measurements were multiplied by eight to determine the collagen content.

#### 2.6.9 Free nucleotides analysis

Free nucleotides were extracted for analysis by using the method described by Guo et al. (2014). Crab muscle samples (approximately 2 g) were homogenized using ultrasonication in 5 mL of 5% TCA and kept on ice for 2 h. The extracts were filtered through a 0.22-μm hydrophilic polyether sulfone (PES) syringe filter (CNW Technologies GmbH, Düsseldorf, Germany) before centrifugation at 10,000 × *g* for 10 min at 4 °C to obtain a clear supernatant. The pellet was extracted once more using 5 mL of 5% TCA as described above. The combined supernatants were adjusted to pH 5.75 and allowed to stabilize for 30 min. The mixture was then diluted to a final volume of 50 mL, filtered through a 0.22-μm membrane filter, and subjected to HPLC (Agilent Technologies Inc., Santa Clara, CA, USA). The gradient, eluent compositions, and HPLC analytical process are all explained in Table S5.

#### 2.6.10 Electronic tongue analysis

Electronic tongue analysis was done using the approach provided by Zeng et al. (2020). Approximately 10 g of muscle tissue was weighed and homogenized with distilled water at a ratio of 1:5 at 40 °C. The supernatant was collected for automated measurement using the TS-5000Z Taste Sensor System (Insent Inc., Atsugi, Japan) after centrifugation at 1200 × *g* for 10 min. This system is equipped with six sensor probes to detect umami and richness, saltiness, sourness, bitterness, astringency, and sweetness. Sensor probes replicate taste perception by measuring membrane potential changes generated by interactions between flavor compounds and artificial lipid membranes. The tasteless calibration solution consisted of 30 mmol/L potassium chloride and 0.3 mmol/L tartaric acid. Each sample underwent four tests, with the first test data discarded and the average of the final three tests used for statistical analysis.

### 2.7 Calculation and statistical analysis

Percentweightgain(PWG,%)=100×(Wt–Wi)/Wi;

Survival(%)=100×Nt/Ni;

Specificgrowthrate(SGR,%/d)=100×(LnWt–LnWi)/t;

Moltingrate(%)=2×Moltingnumber/(Nt+Ni);

Feedefficiency(FE)=Weightgain(g,wetweight)/Feedconsumed(g,dryweight);

Feedintake(FI,%/d)=100×Totalfeedintake(g)/[(Wi/2+Wt/2)×t];

where *t* is the number of experimental days; *Nt* and *Ni* are the final and initial crab numbers; *Wt* and *Wi* are the final and initial body weights, respectively.

The computation of taste activity value (TAV) and equivalent umami concentration (EUC) values was based on earlier studies (Yu et al., 2024; Zhu et al., 2022). The data were shown as means and the pooled standard error of the mean (SEM). As stated in the manuscript, a one-way analysis of variance (ANOVA) was the primary statistical model used to assess the effects of the dietary treatments (the fixed factor with six levels: 0, 10%, 20%, 30%, 40%, and 60% BSFM replacement) on all measured parameters, including growth performance, body composition, flesh quality, gene expression, and biochemical indicators. The model was:

$$Y_{ij}=\mu +\alpha_{i}+\varepsilon_{ij}\text{,}$$

where *Y*_*ij*_ is the observed value; *μ* is the overall mean; *α*_*i*_ is the fixed effect of the *i*-th dietary treatment; and *ε*_*ij*_ is the random error. Prior to ANOVA, all data were rigorously screened to ensure they met the assumptions of normality and homogeneity of variances. Normality was assessed using the Shapiro–Wilk test. Homogeneity of variances was assessed using Levene's test. In this study, the data met the assumption of homogeneity of variance, so no transformation was applied to the data. Following a significant (*P* < 0.05) F-value in ANOVA, post-hoc analyses were conducted to separate means. For ANOVA, the Tukey multiple range test was used for post-hoc comparisons. All statistical analyses were performed using IBM SPSS software version 22.0, and a significance level of *P* < 0.05 is applied throughout the study.

## 3 Results

### 3.1 Safety inspection

As shown in Table 5, no HCH or DDT was detected in the BSFM, and the concentrations of Pb and Cd complied with the requirements specified in the Chinese Feed Hygiene Standards (China National Standard, 2017). The concentrations of Pb in mud crab tissues complied with the maximum levels set by the European Commission Regulation (EC) No 1881/2006. The Cd concentrations were within the limits of the Chinese food safety standard (China National Standard, 2022, Table 6).

**Table 5:** Harmful substance levels (mg/kg) in the black soldier fly larvae meal (based on a wet basis).

Items	Black soldier fly larvae meal	GB 13078–2017 (China National Standard, 2017)
HCH	ND	<0.20
DDT	ND	<0.05
Pb	2.30	<10.00
Cd	0.05	<2.00

HCH = hexachlorocyclohexane; DDT = dichlorodiphenyltrichloroethane; ND = not detected.

**Table 6:** Heavy metal content (mg/kg) in the hepatopancreas and muscle of mud crab fed with different experimental diets for 8 weeks (based on a wet basis).

Items	Replacement of FM with BSFM, %						(EC) No 1881/2006 and GB 2762–2022^1^
	0	10	20	30	40	60	
**Hepatopancreas**							
Pb	0.01	0.018	0.029	0.033	0.038	0.049	<0.050
Cd	0.037	0.073	0.051	0.07	0.076	0.073	<0.100
**Swimming leg muscle**							
Pb	0.043	0.025	0.043	0.025	0.027	0.023	<0.050
Cd	0.044	0.064	0.029	0.056	0.049	0.045	<0.100

FM = fish meal; BSFM = black soldier fly larvae meal.

1 References were European Commission (2006) and China National Standard (2022).

### 3.2 Growth performance and feed utilization

Substituting FM with BSFM had no significant effect on the PWG, SGR, molting ratio, FE, and FI of mud crabs (*P* > 0.05; Table 7). However, the survival of crabs in the BSFM60 group was significantly lower than that in the BSFM0 group (*P* = 0.007).

**Table 7:** Growth performance, feed utilization and survival of mud crabs fed with different experimental diets for 8 weeks.

Items	Replacement of FM with BSFM, %						SEM	*P*-value		
	0	10	20	30	40	60		ANOVA	Linear	Quadratic
IW, g	22.45	22.47	22.45	22.46	22.46	22.49	0.016	0.989	0.644	0.719
PWG, %	107.77	116.94	114.45	101.31	105.06	101.39	3.062	0.609	0.228	0.586
Survival, %	75.00^a^	68.75^ab^	68.75^ab^	75.00^a^	65.63^ab^	59.38^b^	2.128	0.007	0.003	0.078
SGR, %/d	1.31	1.37	1.36	1.25	1.28	1.25	0.026	0.634	0.216	0.626
Molting ratio, %	0.96	0.92	1.03	0.88	1.01	1.05	0.044	0.895	0.573	0.626
FE	0.70	0.64	0.62	0.69	0.68	0.69	0.019	0.852	0.796	0.425
FI, %/d	2.28	2.18	2.36	2.07	2.43	2.48	0.048	0.096	0.100	0.147

FM = fish meal; BSFM = black soldier fly larvae meal; IW = initial weight; PWG = percent weight gain; SGR = specific growth rate; FE = feed efficiency; FI = feed intake; SEM = standard error of the mean.

Within a row, means without a common superscript letter differ at *P* < 0.05, *n* = 4.

### 3.3 Nutritional composition in hepatopancreas and muscle

**Fig. 1:**
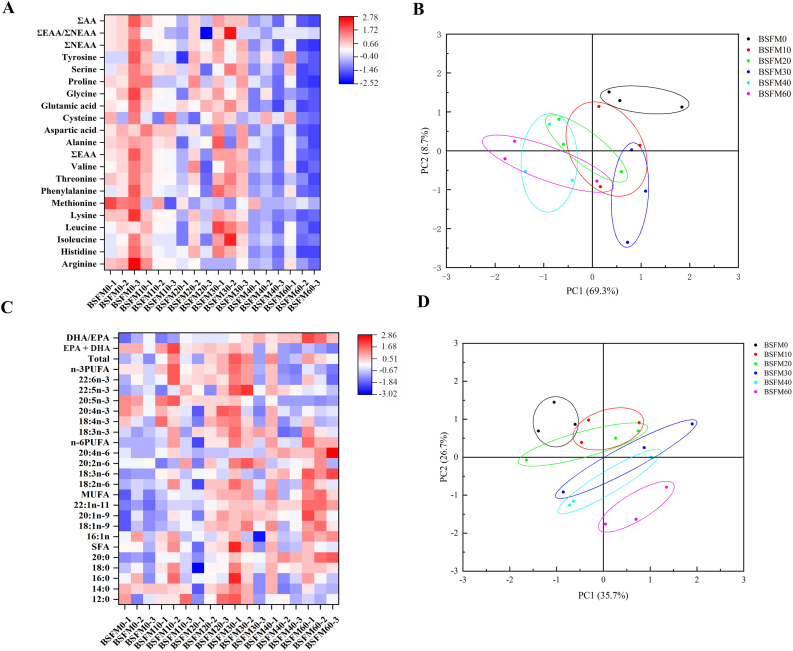
Amino acid and fatty acid compositions in the hepatopancreas of mud crabs fed with different experimental diets for 8 weeks. (A) and (C) Heat maps of the amino acid and fatty acid composition, respectively. (B) and (D) Principal component analysis (PCA) model of the amino acid and fatty acid composition, respectively. BSFM0, BSFM10, BSFM20, BSFM30, BSFM40, and BSFM60, substitution of fish meal with 0, 10%, 20%, 30%, 40%, and 60% black soldier fly larvae meal, respectively. EAA = essential amino acid; NEAA = non-essential amino acid; AA = amino acid; SFA = saturated fatty acids; MUFA = mono-unsaturated fatty acids; PUFA = polyunsaturated fatty acids. Different lowercase letters above columns represent significant differences among treatments at *P* < 0.05 (*n* = 4).

**Fig. 2:**
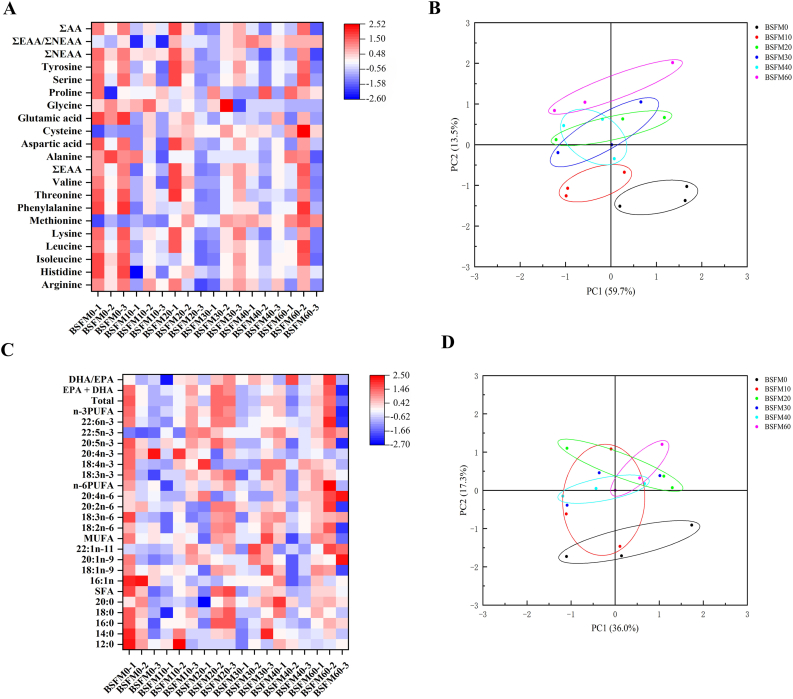
Amino acid and fatty acid compositions in the muscle of mud crabs fed with different experimental diets for 8 weeks. (A) and (C) Heat maps of the amino acid and fatty acid composition, respectively. (B and D) Principal component analysis (PCA) model of the amino acid and fatty acid composition, respectively. BSFM0, BSFM10, BSFM20, BSFM30, BSFM40, and BSFM60, substitution of fish meal with 0, 10%, 20%, 30%, 40%, and 60% black soldier fly larvae meal, respectively. EAA = essential amino acid; NEAA = non-essential amino acid; AA = amino acid; SFA = saturated fatty acids; MUFA = mono-unsaturated fatty acids; PUFA = polyunsaturated fatty acids. Different lowercase letters above columns represent significant differences among treatments at *P* < 0.05 (*n* = 4).

Substituting FM with BSFM did not substantially change the contents of the dry matter, protein, lipid, and ash in hepatopancreas and muscle (*P* > 0.05; Table 8). However, principal component analysis (PCA) and heat map correlation indicated the amino acid profile of hepatopancreas in the BSFM0 group was significantly different from that in other groups and showed a decreasing trend as the level of FM substitution increased (Table S6 and Fig. 1A and B). Crabs fed with BSFM40 and BSFM60 diets showed significantly lower concentrations of arginine, lysine, threonine, glutamic acid, total essential amino acids (ΣEAA), total non-essential amino acids (ΣNEAA), and total amino acids (ΣAA) in the hepatopancreas compared to BSFM0 (*P* < 0.05). Additionally, crabs fed with the BSFM60 diet had markedly reduced histidine, methionine, aspartic acid, glycine, and proline compared to those fed the BSFM0 diet (*P* < 0.05). The BSFM substitution significantly increased the levels of several fatty acids in the hepatopancreas, including 20:0, 18:1n-9, 22:1n-11, 18:3n-6, 20:4n-6 (ARA), and total monounsaturated fatty acids (ΣMUFA) compared to the BSFM0 group (*P* < 0.05). In contrast, 20:5n-3 (EPA) content decreased significantly compared to the BSFM0 group, and other fatty acids remained unchanged (*P* > 0.05; Table S7, Fig. 1C and D). In muscle, increasing BSFM substitution led to significant increases in methionine and cysteine, and a decrease in glutamic acid compared to the BSFM0 group (*P* < 0.05), ΣEAA and ΣNEAA levels remained unchanged (*P* > 0.05; Table S8, Fig. 2A and B). Moreover, the 22:5n-3 fatty acid content significantly increased with higher BSFM substitution compared to the BSFM0 group (*P* = 0.009; Table S9, Fig. 2C and D).

**Table 8:** Proximate composition (%) in muscle and hepatopancreas of mud crabs fed with different experimental diets for 8 weeks (based on wet basis).

Items	Replacement of FM with BSFM, %						SEM	*P-*value		
	0	10	20	30	40	60		ANOVA	Linear	Quadratic
**Hepatopancreas**										
Dry matter	43.22	42.90	42.71	43.16	42.72	42.10	0.424	0.988	0.532	0.802
Protein	14.19	12.59	12.81	13.03	13.82	12.29	0.244	0.162	0.228	0.601
Lipid	20.48	20.86	20.35	21.40	20.59	20.71	0.266	0.931	0.823	0.717
Ash	1.92	1.93	1.94	1.89	1.96	1.82	0.030	0.841	0.493	0.516
**Muscle**										
Dry matter	26.08	26.65	26.78	26.9	26.08	26.28	0.232	0.890	0.906	0.582
Protein	21.29	21.86	22.36	22.41	21.42	21.50	0.224	0.621	0.960	0.136
Lipid	1.55	1.57	1.68	1.74	1.65	1.59	0.043	0.828	0.635	0.266
Ash	2.11	2.14	2.20	2.23	2.15	2.13	0.018	0.418	0.709	0.073

FM = fish meal; BSFM = black soldier fly larvae meal; SEM = standard error of the mean.

Within a row, means are not statistically significant difference at *P* > 0.05, *n* = 4.

### 3.4 Health response

**Fig. 3:**
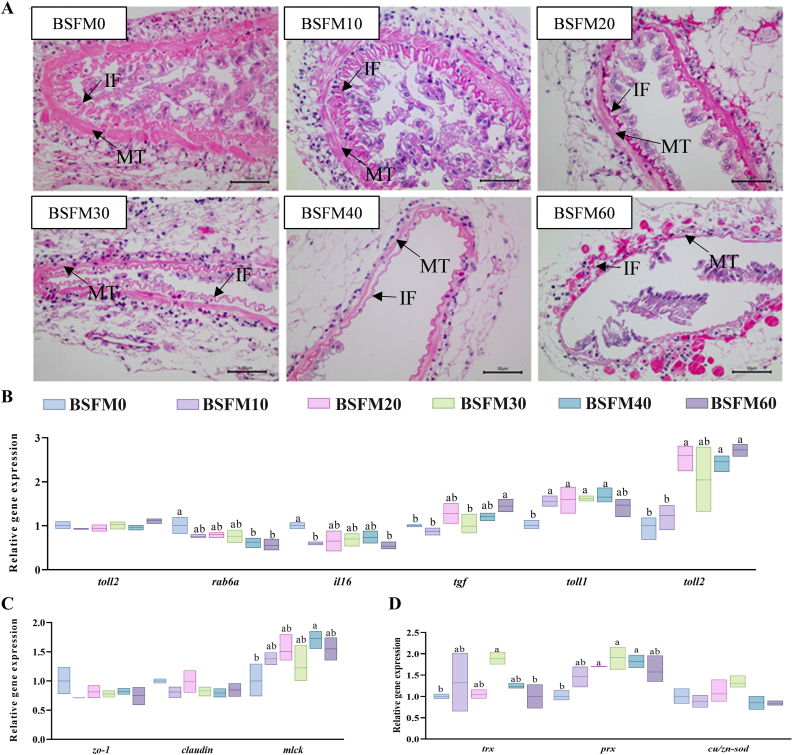
Health response of mud crabs fed with different experimental diets for 8 weeks. (A) Intestinal histological structure (under 200 × magnification). (B) Relative gene expression involved in inflammation in the intestine. (C) Relative gene expression involved in physical barrier in the intestine. (D) Relative gene expression involved in antioxidant in the hepatopancreas. BSFM0, BSFM10, BSFM20, BSFM30, BSFM40, and BSFM60, substitution of fish meal with 0, 10%, 20%, 30%, 40%, and 60% black soldier fly larvae meal, respectively. IF = intestinal folds; MT = muscularis thickness. Different lowercase letters above columns represent significant differences among treatments at *P* < 0.05 (*n* = 4).

Histological analysis of the intestine revealed pronounced alterations in crabs fed with the BSFM60 diet, including reduced length and width of intestinal folds, thinning of the intestinal muscularis, and marked detachment of intestinal villi (Fig. 3A). In parallel, the expression levels of *rab6a* and *il-16* were significantly down-regulated, while those of *tgf*, *toll1*, and *toll2* were markedly up-regulated compared with the BSFM0 group (*P* < 0.05; Fig. 3B). Additionally, the expression of *mlck*, a gene associated with intestinal structural integrity, was markedly up-regulated in those fed with BSFM supplementation (*P* = 0.033). In contrast, no significant variations were seen in the expression levels of claudin and *zo-1* across the dietary treatments (*P* > 0.05; Fig. 3C).

In the hepatopancreas, activities of antioxidant-related enzymes and contents of oxidative stress markers were assessed (Table 9). No notable discrepancies were detected in activities of T-SOD and GSH-Px among the dietary groups (*P* > 0.05). However, the activities of CAT and GSH were markedly elevated in the BSFM30 and BSFM60 groups, respectively (*P* < 0.05). The contents of MDA and PC were not greatly influenced by dietary treatments (*P* > 0.05). At the transcriptional level, mRNA expression of *trx* and *prx* was significantly up-regulated with higher BSFM substitution, peaking in the BSFM30 group (*P* < 0.05). In contrast, mRNA expression of *cu/zn-sod* remained unchanged across all dietary treatments (*P* > 0.05; Fig. 3D).

**Table 9:** Antioxidant indices in hepatopancreas of mud crabs fed with different experimental diets for 8 weeks.

Items	Replacement of FM with BSFM, %						SEM	*P*-value		
	0	10	20	30	40	60		ANOVA	Linear	Quadratic
CAT, U/mg prot	1.43^b^	1.35^b^	1.15^b^	1.33^b^	2.63^a^	2.61^a^	0.159	<0.001	<0.001	0.001
T-SOD, U/mg prot	48.58	45.34	46.13	48.85	48.62	49.06	1.075	0.908	0.556	0.607
GSH, mg/g prot	45.85^c^	51.96^bc^	53.31^bc^	66.75^a^	58.41^ab^	52.41^bc^	1.804	0.002	0.010	0.001
GSH-Px, U/mg prot	233.01	244.50	246.00	252.67	256.37	241.95	6.460	0.953	0.576	0.480
MDA, nmol/mg prot	1.14	1.13	0.97	0.98	0.96	1.07	0.034	0.510	0.250	0.186
PC, nmol/mg prot	6.70	6.15	6.58	6.45	7.12	6.20	0.156	0.552	0.936	0.813

FM = fish meal; BSFM = black soldier fly larvae meal; CAT = catalase; ptot = protein; T-SOD = superoxide dismutase; GSH = glutathione; GSH-Px = glutathione peroxidase; MDA = malondialdehyde; PC = protein carbonyl; SEM = standard error of the mean.

Within a row, means without a common superscript letter differ at *P* < 0.05, *n* = 4.

### 3.5 Morphology and physical properties

**Fig. 4:**
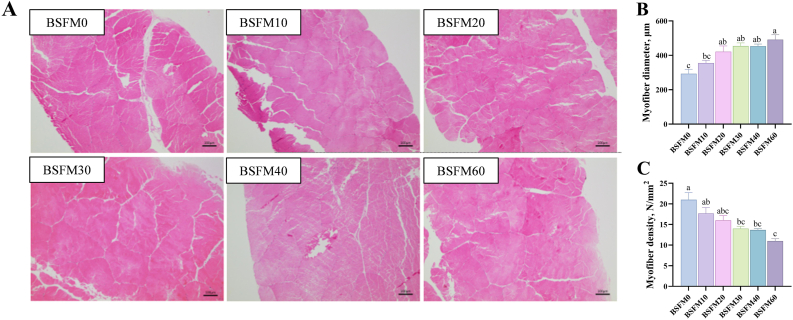
Morphology and physical properties in the muscle of mud crabs fed with different experimental diets for 8 weeks. (A) Muscle morphology (under 100 × magnification). (B) Diameter of myofiber. (C) Density of myofibers (random 10 myofibers of every section, three sections of every group). BSFM0, BSFM10, BSFM20, BSFM30, BSFM40, and BSFM60, substitution of fish meal with 0, 10%, 20%, 30%, 40%, and 60% black soldier fly larvae meal, respectively. Different lowercase letters above columns represent significant differences among treatments at *P* < 0.05 (*n* = 4).

Muscle morphology of mud crabs is shown in Fig. 4A. With the increase of replacement of FM with BSFM, myofiber diameter significantly increased, while muscle fiber density decreased (*P* < 0.05; Fig. 4B and C). As shown in Table 10, textural parameters, including hardness, springiness, chewiness, and gumminess, were significantly enhanced with increasing BSFM substitution compared to the BSFM0 group (*P* < 0.05). However, resilience and cohesiveness remained unaffected by dietary treatments (*P* > 0.05). No significant differences were seen in pH, water loss, or lipid loss among the dietary groups (*P* > 0.05). Notably, liquid loss was significantly reduced in crabs fed BSFM-substituted diets compared to the BSFM0 group (*P* = 0.007). After three days of storage at 4 °C, muscle pH values increased progressively with higher levels of BSFM substitution, indicating a slower postmortem pH decline (*P* = 0.039). In addition, muscle collagen content was substantially elevated in the BSFM30, BSFM40, and BSFM60 groups compared to the BSFM0 group (*P* = 0.013).

**Table 10:** Physical properties in the muscle of mud crabs fed with different experimental diets for 8 weeks.

Items	Replacement of FM with BSFM, %						SEM	*P*-value		
	0	10	20	30	40	60		ANOVA	Linear	Quadratic
Hardness, g	67.03^c^	74.06^bc^	73.65^bc^	76.95^a^	74.17^bc^	79.43^a^	1.089	0.005	0.001	0.003
Springiness, mm	0.41^c^	0.49^ab^	0.46^abc^	0.46^abc^	0.44^bc^	0.51^a^	0.009	0.002	0.064	0.186
Chewiness, g	11.92^b^	14.00^ab^	14.18^ab^	15.84^ab^	14.23^ab^	16.23^a^	0.451	0.048	0.005	0.016
Gumminess, g	25.60^c^	32.10^b^	28.24^bc^	32.29^b^	33.37^b^	41.89^a^	1.303	<0.001	<0.001	<0.001
Cohesiveness	0.47	0.49	0.49	0.49	0.46	0.48	0.005	0.476	0.782	0.749
Resilience, N	0.14	0.14	0.14	0.15	0.14	0.15	0.003	0.900	0.481	0.787
pH (0 d)	6.79	6.82	6.80	6.79	6.81	6.83	0.008	0.572	0.238	0.428
pH (3 d)	5.90^b^	5.94^ab^	6.13^ab^	6.04^ab^	6.13^ab^	6.23^a^	0.037	0.039	0.002	0.008
Liquid loss, %	29.91^a^	27.56^ab^	25.73^ab^	24.64^b^	24.10^b^	25.10^b^	0.578	0.007	0.001	<0.001
Water loss, %	20.35	20.22	19.26	19.56	19.48	20.69	0.470	0.962	0.985	0.654
Lipid loss, %	4.95	5.45	4.64	5.08	5.20	5.33	0.118	0.470	0.540	0.663
Collagen, mg/g	1.39^b^	1.53^ab^	1.51^ab^	1.62^a^	1.60^a^	1.59^a^	0.023	0.013	0.002	0.002

FM = fish meal; BSFM = black soldier fly larvae meal; SEM = standard error of the mean.

Within a row, means without a common superscript letter differ at *P* < 0.05, *n* = 4.

### 3.6 Non-volatile taste substances

The concentrations of 20 FAAs in crab muscle are summarized in Table 11, with their corresponding TAVs calculated and presented in Table 12. The levels of serine, glycine, alanine, isoleucine, and leucine exhibited an initial increase followed by a decrease as the substitution level of BSFM increased, especially reaching the lowest value in the BSFM60 group (*P* < 0.05). Notably, the concentration of umami free amino acids (UFAA) was substantially diminished in the BSFM60 group compared to the BSFM0 group, showing a negative correlation with increasing BSFM substitution (*P* = 0.002). Similarly, the contents of sweet FAAs (SFAA) and bitter FAAs (BFAA) also displayed an initial rise and subsequent decline with the increase of the substitution level of BSFM, and the highest value was obtained in the BSFM20 group (*P* < 0.05). In contrast, no substantial changes were seen in the delightful FAAs (DFAA) and total FAAs (TFAA) among the dietary groups (*P* > 0.05).

**Table 11:** Free amino acid (FAA) contents (mg/100 g) of the muscle of mud crabs fed with different experimental diets for 8 weeks (wet basis)^1^.

FAAs	Taste^2^	Replacement of FM with BSFM, %						SEM	*P*-value		
		0	10	20	30	40	60		ANOVA	Linear	Quadratic
Aspartic acid	Umami (+)	11.79	12.15	12.19	12.04	11.15	11.17	0.181	0.353	0.107	0.194
Glutamic acid	Umami (+)	28.75	29.57	28.59	29.18	27.35	27.08	0.336	0.184	0.038	0.215
Serine	Sweet (+)	4.21^ab^	5.96^ab^	5.62^ab^	6.73^a^	4.75^ab^	3.07^b^	0.372	0.027	0.171	0.002
Glycine	Sweet (+)	418.28^b^	519.14^ab^	544.94^a^	496.02^ab^	505.56^ab^	480.03^ab^	12.194	0.027	0.260	0.005
Threonine	Sweet (+)	14.72	16.14	15.45	17.47	16.66	15.55	0.292	0.063	0.135	0.029
Alanine	Sweet (+)	141.53^b^	171.30^a^	173.39^a^	163.16^a^	166.31^a^	145.49^b^	3.325	0.001	0.883	<0.001
Proline	Sweet (+)	301.88	306.34	339.68	337.39	323.52	346.74	9.413	0.726	0.210	0.682
Histidine	Bitter (−)	15.35	16.17	17.17	17.32	15.85	15.87	0.250	0.114	0.690	0.013
Arginine	Bitter (−)	332.70	337.23	339.39	330.88	318.75	294.42	5.319	0.106	0.017	0.070
Tyrosine	Bitter (−)	28.06	32.33	32.54	30.25	28.74	27.67	0.692	0.139	0.249	0.029
Valine	Bitter (−)	29.05	29.81	33.42	33.23	29.23	29.53	0.677	0.165	0.972	0.032
Methionine	Bitter (−)	27.00	30.60	26.54	27.22	27.03	23.86	0.882	0.465	0.180	0.372
Tryptophan	Bitter (−)	4.46	5.02	5.64	6.105	4.63	5.07	0.222	0.266	0.587	0.079
Phenylalanine	Bitter (−)	16.50	17.59	18.28	17.12	17.22	16.04	0.237	0.063	0.270	0.008
Isoleucine	Bitter (−)	13.31^bc^	14.75^a^	15.07^a^	14.60^ab^	13.18^bc^	12.57^b^	0.254	0.001	0.008	<0.001
Leucine	Bitter (−)	23.52^b^	28.44^a^	28.94^a^	29.04^a^	27.78^a^	24.76^b^	0.580	0.001	0.502	<0.001
Lysine	Bitter (−)	12.10	14.93	14.79	15.66	14.64	14.45	0.360	0.055	0.068	0.013
Asparagine	Tasteless	23.97	28.70	29.96	29.56	30.25	25.51	1.001	0.361	0.559	0.042
Cysteine	Tasteless	0.70	0.68	0.68	0.85	0.77	0.63	0.038	0.690	0.911	0.328
Glutamine	Tasteless	196.58	223.15	220.52	230.77	230.17	193.92	7.665	0.633	0.914	0.115
UFAA	Umami (+)	40.53^ab^	41.72^a^	40.78^a^	41.21^a^	38.50^bc^	38.25^c^	0.367	0.002	0.001	0.012
SFAA	Sweet (+)	880.63	1018.87	1079.07	1020.76	1016.79	990.88	19.899	0.069	0.167	0.012
BFAA	Bitter (−)	502.05^ab^	526.86^a^	531.76^a^	521.41^a^	497.06^ab^	464.24^b^	6.927	0.017	0.014	0.003
DFAA		1423.21	1587.44	1651.60	1583.38	1552.34	1493.37	23.702	0.052	0.652	0.004
TFAA		1644.45	1839.97	1902.77	1844.56	1813.54	1713.44	29.121	0.074	0.678	0.005

FM = fish meal; BSFM, black soldier fly larvae meal; UFAA = umami free amino acids; SFAA = sweet free amino acids; BFAA = bitter free amino acids; DFAA = delicious free amino acids; TFAA = total free amino acids; SEM = standard error of the mean.

Within a row, means without a common superscript letter differ at *P* < 0.05, *n* = 4.

1 The type of amino acid and associated flavor refer to Yu et al. (2024).

2 (+), pleasant; (−), unpleasant.

**Table 12:** Taste activity values (TAVs, mg/100 g) of free amino acids (FAAs) in the muscle of mud crabs fed with different experimental diets for 8 weeks (wet basis)^1^.

FAAs	Taste^2^	Taste threshold, mg/100 mL	Replacement of FM with BSFM, %						SEM	*P*-value		
			0	10	20	30	40	60		ANOVA	Linear	Quadratic
Aspartic acid	Umami (+)	100	0.12	0.12	0.12	0.12	0.11	0.11	0.002	0.381	0.129	0.203
Glutamic acid	Umami (+)	30	0.96	0.99	0.95	0.97	0.91	0.90	0.011	0.185	0.039	0.213
Serine	Sweet (+)	150	0.03^ab^	0.04^ab^	0.04^ab^	0.05^a^	0.03^ab^	0.02^b^	0.003	0.023	0.154	0.002
Glycine	Sweet (+)	130	3.22^b^	3.99^ab^	4.19^a^	3.82^ab^	3.89^ab^	3.69^ab^	0.094	0.027	0.259	0.005
Threonine	Sweet (+)	260	0.06	0.06	0.06	0.07	0.06	0.06	0.001	0.058	0.109	0.031
Alanine	Sweet (+)	60	2.36^b^	2.86^a^	2.89^a^	2.72^a^	2.77^a^	2.43^b^	0.055	0.001	0.886	<0.001
Proline	Sweet (+)	300	1.01	1.02	1.13	1.12	1.08	1.16	0.031	0.725	0.210	0.683
Histidine	Bitter (−)	20	0.77	0.81	0.86	0.87	0.79	0.79	0.012	0.114	0.697	0.013
Arginine	Bitter (−)	50	6.65	6.74	6.79	6.62	6.37	5.89	0.106	0.106	0.017	0.070
Tyrosine	Bitter (−)	–	–	–	–	–	–	–	–			
Valine	Bitter (−)	40	0.73	0.75	0.84	0.83	0.73	0.74	0.017	0.166	0.969	0.032
Methionine	Bitter (−)	30	0.90	1.02	0.88	0.91	0.90	0.80	0.029	0.464	0.180	0.372
Tryptophan	Bitter (−)	–	–	–	–	–	–	–	–			
Phenylalanine	Bitter (−)	90	0.18	0.20	0.20	0.19	0.19	0.18	0.003	0.065	0.280	0.008
Isoleucine	Bitter (−)	90	0.15^bcd^	0.16^ab^	0.17^a^	0.16^abc^	0.15^cd^	0.14^d^	0.003	0.001	0.009	<0.001
Leucine	Bitter (−)	190	0.12^b^	0.15^a^	0.15^a^	0.15^a^	0.15^a^	0.13^b^	0.003	<0.001	0.492	<0.001
Lysine	Bitter (−)	50	0.24	0.30	0.30	0.31	0.29	0.29	0.007	0.055	0.067	0.014
Asparagine	Tasteless	–	–	–	–	–	–	–	–	–	–	–
Cysteine	Tasteless	–	–	–	–	–	–	–	–	–	–	–
Glutamine	Tasteless	–	–	–	–	–	–	–	–	–	–	–

FM = fish meal; BSFM = black soldier fly larvae meal; SEM = standard error of the mean.

Within a row, means without a common superscript letter differ at *P* < 0.05, *n* = 4.

1 The type of amino acid and associated flavor refer to Yu et al. (2024).

2 (+), pleasant; (−), unpleasant; -, not be determined.

**Fig. 5:**
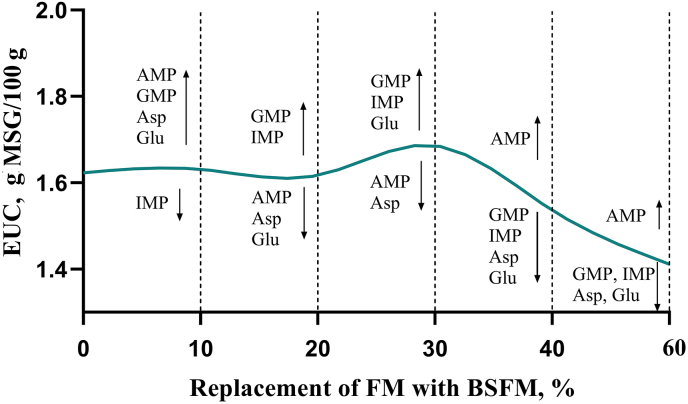
The trend of equivalent umami concentration (EUC) values in the muscle of mud crabs fed with different experimental diets for 8 weeks. Arrows indicated the trend of EUC values in muscle tissue with the change of umami substances. FM = fish meal; BSFM = black soldier fly larvae meal; AMP = adenosine monophosphate; GMP = guanosine monophosphate; IMP = inosine monophosphate; MSG = monosodium glutamate. ↑ = increased content with increasing replacement level; ↓ = decreased content with increasing replacement level.

Data on flavor nucleotide contents are presented in Table S9, with their corresponding TAVs calculated and presented in Table 13. No substantial changes were seen in adenosine monophosphate (AMP) and inosine monophosphate (IMP) TAV among the experimental groups (*P* > 0.05). In contrast, the TAV of guanosine monophosphate (GMP) in crab muscle first increased and then decreased across the dietary treatments, reaching its lowest level in the BSFM60 group (*P* = 0.004). However, the concentration of hypoxanthine (Hx), a degradation product of IMP associated with bitter taste, in the BSFM replacement group was significantly higher than that in the control group (*P* = 0.020). Integrating the contributions of FAAs and free nucleotides, the EUC of crab muscle was reduced in the BSFM40 and BSFM60 groups, with BSFM60 achieving the lowest value (*P* = 0.019; Fig. 5).

**Table 13:** Taste activity values (TAVs, mg/100 g) of flavor nucleotides in the muscle of mud crabs fed with different experimental diets for 8 weeks (wet basis)^1^.

Flavor nucleotides	Taste^2^	Taste threshold, mg/100 mL	Replacement of FM with BSFM, %						SEM	*P*-value		
			0	10	20	30	40	60		ANOVA	Linear	Quadratic
AMP	Umami/sweet (+)	50	1.68	1.78	1.44	1.25	1.35	1.41	0.067	0.153	0.037	0.266
GMP	Umami (+)	12.5	0.43^b^	0.45^ab^	0.49^a^	0.50^a^	0.44^ab^	0.41^b^	0.009	0.004	0.399	<0.001
IMP	Umami (+)	25	10.20	10.11	10.40	10.76	10.19	9.47	0.158	0.323	0.342	0.076
Hx	Bitter (−)	–	9.72^b^	13.88^a^	12.93^ab^	14.02^a^	13.98^a^	12.89^ab^	0.458	0.020	0.026	0.010
EUC			1.62^ab^	1.63^ab^	1.62^ab^	1.69^a^	1.54^ab^	1.41^b^	0.027	0.019	0.008	0.017

FM = fish meal; BSFM = black soldier fly larvae meal; AMP = adenosine monophosphate; GMP = guanosine monophosphate; IMP = inosine monophosphate; Hx = hypoxanthine; EUC = equivalent umami concentration; SEM = standard error of the mean.

Within a row, means without a common superscript letter differ at *P* < 0.05, *n* = 4.

1 The type of amino acid and associated flavor refer to Yu et al. (2024).

2 (+), pleasant; (−), unpleasant; -, not be determined.

### 3.7 Flesh electronic tongue and PCA

**Fig. 6:**
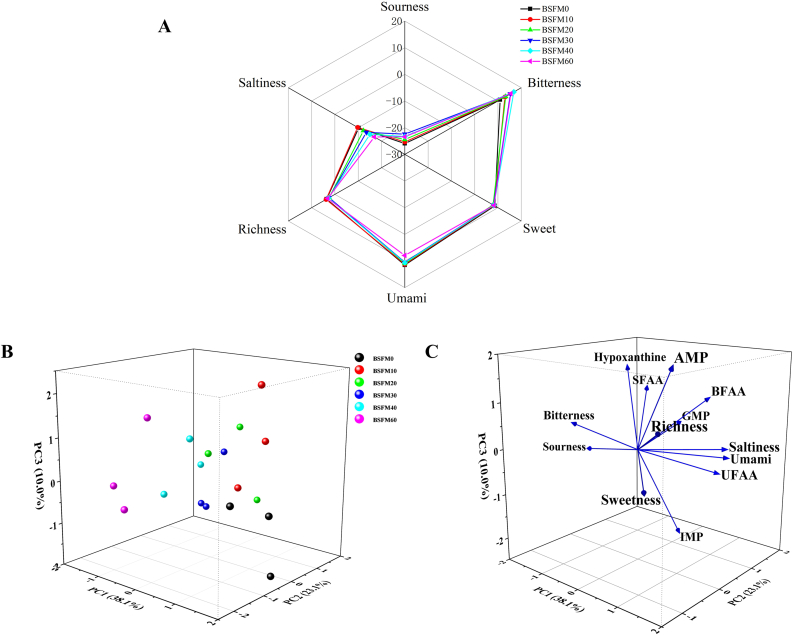
Flesh electronic tongue and principal component analysis of mud crabs fed with different experimental diets for 8 weeks (*n* = 4). (A) Radar image analysis of muscle electronic tongue. The sign and magnitude of the values indicate the relative strength of each taste attribute. (B and C) Principal component analysis (PCA) of flavor substances (free amino acids, flavor nucleotide, and electronic tongue). BSFM0, BSFM10, BSFM20, BSFM30, BSFM40, and BSFM60, substitution of fish meal with 0, 10%, 20%, 30%, 40%, and 60% black soldier fly larvae meal, respectively. UFAA = umami free amino acids; SFAA = sweet umami free amino acids; BFAA = bitter free amino acids; AMP = adenosine monophosphate; GMP = guanosine monophosphate; IMP = inosine monophosphate; PC = principal component.

Figure 6A illustrates the sensor response values of muscle samples from six dietary groups, as measured by the electronic tongue across six distinct taste sensors. Overall, the umami, sweetness, and bitterness sensors exhibited higher response values across all samples, suggesting these were the predominant taste attributes. As shown in Table 14, umami, sweetness, and saltiness intensities in crab muscle declined significantly with increased BSFM substitution (*P* < 0.05). Furthermore, the richness perception also decreased markedly, whereas sourness and bitterness values increased considerably in the BSFM20 and BSFM30 groups compared to the BSFM0 group (*P* < 0.05).

**Table 14:** Taste profiles of electronic tongue in the muscle of mud crabs fed with different experimental diets for 8 weeks.

Items	Replacement of FM with BSFM, %						SEM	*P*-value		
	0	10	20	30	40	60		ANOVA	Linear	Quadratic
Sourness	−25.94^c^	−25.35^c^	−24.46^bc^	−22.42^a^	−23.27^ab^	−23.41^ab^	0.318	<0.001	<0.001	0.004
Bitterness	10.95^c^	13.01^b^	13.31^b^	15.36^a^	16.81^a^	15.55^a^	0.494	<0.001	<0.001	0.009
Sweetness	8.62^a^	8.31^ab^	7.94^c^	8.11^bc^	8.21^bc^	8.26^bc^	0.056	<0.001	0.008	<0.001
Umami	11.51^a^	11.22^ab^	10.93^ab^	10.48^b^	10.66^b^	7.92^c^	0.293	<0.001	<0.001	<0.001
Richness	3.57^a^	3.78^a^	2.47^b^	2.48^b^	2.76^ab^	3.08^ab^	0.144	0.004	0.011	0.004
Saltiness	−10.20^a^	−9.78^a^	−12.24^ab^	−13.48^ab^	−15.02^ab^	−16.92^b^	0.780	0.025	0.001	0.549

FM = fish meal; BSFM = black soldier fly larvae meal; SEM = standard error of the mean.

Within a row, means without a common superscript letter differ at *P* < 0.05, *n* = 4.

The PCA was performed based on sensory attributes and non-volatile taste compounds, and the results are visualized in the three-dimensional score and loading plots (Fig. 6B and C). The analysis revealed clear differentiation among the six dietary groups. Principal components (PC) 1, 2, and 3 accounted for 38.1%, 23.1%, and 10.0% of the total variance, respectively. The distribution of samples in the score plot demonstrated that increasing levels of BSFM substitution were positively associated with bitterness and sourness, while negatively correlated with umami, saltiness, UFAA, AMP, GMP, IMP, richness, SFAA, and sweetness. The loading plot (Table S10 and Fig. 6C) further confirmed these relationships. Specifically, UFAA, AMP, GMP, and IMP were positively associated with umami perception but negatively correlated with bitterness and sourness. Bitter free amino acids showed a positive correlation with Hx, yet a negative association with bitterness. Additionally, IMP exhibited a positive relationship with sweetness, whereas SFAA was negatively correlated with this attribute.

## 4 Discussion

### 4.1 Safety and contaminant risk assessment

The safety of insect-based feed ingredients represents a primary concern for their application in aquaculture. In this study, no detectable residues of OCPs, including HCH and DDT, were found in BSF larvae. Additionally, the concentrations of Cd and Pb in the hepatopancreas and muscle tissues of both BSF larvae and the experimental crabs fed with BSF larvae for eight weeks were below the maximum limits established by the European Commission Regulation (EC) No 1881/2006 and Chinese regulatory standards (European Commission, 2006; Georgescu et al., 2020). This indicates that using BSFM instead of FM does not pose a risk of heavy metal or pesticide contamination to mud crabs. This might be because the BSF larvae utilized in this experiment were fed on kitchen trash—materials either originally intended for human consumption or derived as food by-products—thereby ensuring a hygienic rearing environment. These findings underscore the critical importance of substrate quality control in BSFM production, aligning with previous research emphasizing the role of rearing substrates in determining insect meal safety (Navajas-Porras et al., 2024; Ganesan et al., 2024).

### 4.2 Growth performance and survival

The growth performance and feed utilization of mud crabs were unaffected in the current research when up to 60% of FM was substituted with BSFM in the diet (300 g/kg FM in the baseline diet). This result is in line with earlier research on BSFM usage in fish diets, which found no detrimental effects on feed intake or growth performance (Belghit et al., 2019; Monteiro dos Santos et al., 2022; Tippayadara et al., 2021; Xu et al., 2020). Given the critical role that dietary protein quality plays in animal development, mud crabs' effective use of BSFM may be explained by its reasonably well-balanced amino acid composition and excellent palatability. However, it is noteworthy that in studies involving yellow catfish (*Pelteobagrus fulvidraco*) and barramundi (*Lates calcarifer*), excessive replacement of FM with BSFM (exceeding 85% and 50%, respectively) resulted in reduced growth performance (Katya et al., 2017; Xiao et al., 2018). These findings highlighted species-specific responses to BSFM-based diets, likely influenced by both variations in the nutrient composition of BSF larvae (driven by differences in rearing substrates) and by the species-specific capacities to digest and utilize BSFM. Crabs given the BSFM60 diet in this research did not exhibit any appreciable differences in growth performance when compared to those on the BSFM0 diet; nevertheless, their survival rate was noticeably lower than that of the BSFM0 group. This might also be related to the fact that as the addition level of BSFM increases, the water stability of the experimental feed decreases (Eide et al., 2024). Mortality can affect the estimation of feed efficiency, as the feed consumed by crabs that died during the trial was included in total FI but did not contribute to overall biomass gain. Consequently, this may lead to a slight underestimation of both PWG and FE. A potential concern in feeding trials is the leaching of nutrients from uneaten feed, which could confound the interpretation of growth performance. In the present study, however, the recorded amount of leftover feed was consistently minimal (ranging from 3.55% to 8.78% of the feed offered) and exhibited no correlation with the level of BSFM inclusion. This indicates high palatability across all feeds and, crucially, minimized both the duration and extent of nutrient leaching. Furthermore, the absence of a systematic increase in leftovers with higher BSF meal levels suggests that any minor leaching would have been uniform across dietary treatments and thus would not introduce a systematic bias in the comparison of experimental results. Therefore, the observed effects on mud crab growth and feed utilization can be robustly attributed to the dietary formulations themselves. In the study, while mortality may have led to a minor underestimation of performance indices, its influence was limited and did not alter the overall conclusions. Interestingly, another study evaluating the replacement of FM with enzymatically hydrolyzed BSFM in mud crabs (using a 40% basal FM diet) reported that survival and weight gain rates significantly increased with rising replacement levels (0–15%) compared to the control group (Yang et al., 2023). This phenomenon could be attributed to the presence of bioactive peptides in the hydrolyzed protein, which may enhance the digestibility and utilization of BSFM. However, the high basal FM level of 40% could also be a contributing factor to the positive outcomes observed. Therefore, the level of base FM in the formula is also a key factor influencing the substitution results. Deficiency of essential fatty acids in the diet or fatty acid imbalances can frequently lead to slower growth and increased mortality (Yang et al., 2025). The basal FM level in this study was set at 300 g/kg, consistent with the level reported to support optimal growth in mud crabs (Deng et al., 2025; Peng et al., 2025; Tang et al., 2024). Therefore, the growth performance and survival rates achieved by replacing FM with BSFM at this basal level are considered valid and consistent with published data (Xu et al., 2024; Zhang et al., 2025). The high and comparable mortality observed in this study is not uncommon for this species under captive culture conditions, particularly during the sensitive molting stages. It has been reported that under different nutritional conditions, the survival rate of crabs ranges from 40% to 85%, with control groups typically showing survival rates between 70% and 85% (Li et al., 2023b; Yuan et al., 2019; Yang et al., 2024). In general, the selection of smaller crabs, especially domesticated crabs, has a higher survival rate. No abnormalities were observed in the experimental conditions or throughout the course of the experiment. Compared with the reported crabs with an initial weight of 1.00 to 10.00 g, the crabs with an initial weight of 22.50 g had a larger body size, longer molting time, and higher mortality after molting. Under the conditions of this experiment, it is reasonable and reliable that the influence range of different BSFM replacing FM on the survival rate of hairy crabs is 59.38% to 75.00%. The growth performance and survival in this study were within reasonable limits from published data on similar species (Holme et al., 2007; Huang et al., 2024; Wang et al., 2024; Yang et al., 2025).

### 4.3 Nutritional composition in hepatopancreas and muscle

This study demonstrates that substituting up to 60% of FM with BSFM in diets for mud crabs effectively maintains the proximate composition and muscle amino acid profile. The primary metabolic response to high BSFM inclusion was a significant reduction in most amino acids within the hepatopancreas. Muscle and hepatopancreas are the crab's main edibles. The protein, amino acids, and unsaturated fatty acids in crustaceans' edible parts determine their nutritional value for consumers (Hu et al., 2023; Yuan et al., 2020). For aquaculture animals in limited conditions, feed quality is the main source of nutrients and energy (Long et al., 2024). The proximate composition of the whole body often reflects this. In this study, replacing up to 60% of FM with BSFM in mud crab diets did not affect the proximate composition in muscle or hepatopancreas. *Channa striata* and *Colossoma macropomum* showed similar results (Monteiro Dos Santos et al., 2023; Siddaiah et al., 2023). Protein synthesis requires amino acids. The amino acid composition of tissues and feed is usually positively correlated. When BSFM replaced over 30% of FM in the diet, the hepatopancreas' amino acid profile demonstrated a significant decrease in most individual amino acids, ΣEAA, ΣNEAA, and ΣAA levels. Hepatopancreatic amino acid composition followed dietary trends and was consistent with previous findings in African catfish, where barley protein partially replaced FM (Zaretabar et al., 2021). In crabs fed BSFM-based diets, crystalline methionine and lysine were supplemented to match the control diet, but assimilation was less efficient. A key factor is the asynchrony in absorption between free crystalline amino acids and protein-bound amino acids from BSFM. The presence of chitin and other structural components in BSFM may have also played a role. While chitin may offer prebiotic benefits, at higher inclusion levels, it can physically encapsulate nutrients and increase the speed of digesta passage, potentially reducing the overall digestibility of protein and the absorption efficiency of all amino acids, including the supplemented ones. This could contribute to the observed downward trend in hepatopancreas amino acids with increasing BSFM substitution. However, the muscle concentrations of most amino acids (ΣEAA, ΣNEAA, and ΣAA) were not significantly affected. The BSFM inclusion increased muscle methionine and cysteine levels unexpectedly. Nutrition and intricate metabolic control in the crab altered the amino acid content of the hepatopancreas and muscle. The hepatopancreas' metabolic role in nutrient absorption, export, and de novo synthesis may explain this discrepancy (Kari et al., 2022). The non-reproductive period of crabs, when dietary amino acid availability declines, may cause muscle tissue to receive more amino acids for somatic growth. Therefore, the significant decrease in EAAs in the hepatopancreas reflects a metabolic trade-off rather than a simple dietary deficiency. The considerable maintenance of EAAs in the muscle, in contrast, indicates the presence of a metabolic prioritization phenomenon within the organism. Further research is needed to understand the mechanisms. The FAO/WHO proposed an ideal protein standard in 1973, recommending ΣEAA/ΣAA and ΣEAA/ΣNEAA ratios of 40% and 60% or higher (Hu et al., 2023). The study found that hepatopancreas and muscle of mud crabs had ΣEAA/ΣAA ratios of around 46%, and ΣEAA/ΣNEAA ratios exceeded 80%, aligning with the FAO/WHO ideal protein profiles.

This study reveals that the substitution of FM with BSFM in mud crab diets exerts a tissue-specific influence on fatty acid profiles. While the hepatopancreas showed a significant marked reduction in EPA, the muscle fatty acid composition remained largely stable. Fatty acids are essential for energy synthesis, growth, and a variety of metabolic functions in animals (Tocher et al., 2019). Marine crustaceans, rich in long-chain polyunsaturated fatty acids (LC-PUFAs) such as EPA and DHA, provide significant health benefits to humans. In the present study, dietary BSFM substitution significantly elevated the concentrations of 20:0, 18:1n-9, 22:1n-11, 18:3n-6, arachidonic acid (ARA), and ΣMUFA in the hepatopancreas. Crabs fed with the BSFM60 diet exhibited a marked reduction in EPA concentration and a tendency toward an increased DHA/EPA ratio, consistent with findings reported in rainbow trout (*Oncorhynchus mykiss*) (Mancini et al., 2018). These findings indicated that dietary BSFM supplementation had a substantial effect on the fatty acid content of mud crabs' hepatopancreas. However, there were no significant changes in muscle fatty acid concentrations, except for a considerable rise in 22:5n-3 levels. The alterations in fatty acid composition in both the hepatopancreas and muscle may be related to the increasing BSFM inclusion in the diet. This may be due to the influence of certain specific fatty acids, such as lauric acid (12:0), present in BSFM, which could have affected fatty acid metabolism, leading to changes in the fatty acid composition of both the hepatopancreas and muscle. The specific mechanisms underlying this relationship, however, require further investigation. The relatively low concentration of lauric acid in mud crab muscle, compared to the hepatopancreas, suggests that 12:0 is more readily oxidized in the muscle for energy production rather than accumulating. Short- and medium-chain fatty acids may enter mitochondria for energy metabolism via passive diffusion, whereas long-chain fatty acids require carnitine-assisted transport. Therefore, the catabolism of 12:0 is more efficient for energy generation (Liu et al., 2021). In conclusion, modest amounts of BSFM (10%–30%) used as a replacement for FM had no significant effect on the quality of amino acids and fatty acids in mud crab hepatopancreas and muscle. However, larger levels of BSFM replacement (40%–60%) resulted in lower amino acid levels in the hepatopancreas, as well as lower EPA concentration.

### 4.4 Health response and antioxidant capacity

The findings in mud crabs demonstrate a dose-dependent effect of dietary BSFM on intestinal health and immunity. Moderate inclusion of BSFM exerted beneficial immunomodulatory effects, whereas a high substitution level (60%) induced significant intestinal structural damage and increased mortality. These results underscore the dual role of BSFM, as a functional immunomodulator at appropriate levels and a potential stressor when in excess. The immunomodulatory capacity of BSFM is likely linked to its chitin content. It is widely recognized that dietary chitin, at appropriate levels, can modulate gut microbiota composition, enhance antioxidant and immune responses, and improve nutrient absorption in animals (Tran et al., 2024). In the present study, BSFM-based diets down-regulated the expression levels of genes associated to pro-inflammatory mediators such as *rab6a* and *il-16*, while up-regulating the expression of genes related to anti-inflammatory cytokines such as *tgf*, *toll1*, and *toll2* in the intestine. These findings indicated that BSFM may function as an immunomodulator by enhancing gut immune function through the suppression of pro-inflammatory mediators and the activation of anti-inflammatory responses, a phenomenon consistent with reports in other aquatic species. For instance, the substitution of FM with 5%–10% BSFM did not compromise the health or disease resistance of the Pacific white shrimp (*Litopenaeus vannamei*). Moreover, all BSFM-fed groups exhibited significantly higher survival rates than the control group following the bacterial challenge (Keetanon et al., 2024). Recent research has demonstrated that BSFM may partially replace FM, enhancing antioxidant and immune functions in several fish species, including Nile tilapia (*Oreochromis niloticus*), snakehead (*C*. *striata*), and grass carp (*Ctenopharyngodon idellus*) (Li et al., 2023c; Siddaiah et al., 2023; Tippayadara et al., 2021). Therefore, dietary components such as chitin-rich BSFM may influence host immunity by modulating the expression of these key immune factors and signaling pathways. Similarly, low quantities of dietary chitin have also been demonstrated to boost the immune system in gilthead seabream (*Sparus aurata* L.) and European sea bass (*Dicentrarchus labrax*) via immunological activation (Esteban et al., 2001; Henry et al., 2018). However, this beneficial effect appears to be limited to diets with low chitin content. In this study, crabs fed the BSFM60 diet exhibited intestinal damage characterized by reduced length and width of intestinal folds and decreased thickness of the intestinal musculature. Intestinal health largely depends on the integrity of the physical barrier (Xiong et al., 2018), which is maintained through intercellular junctions and structural proteins, particularly tight junctions and peritrophic proteins. Notably, the function of tight junctions was negatively associated with expression of *mlck*, indicating that up-regulation of the expression level of *mlck* may impair tight junction integrity (Zhang et al., 2024). Moreover, BSFM diets up-regulated the expression of *mlck* without significantly affecting the expression levels of *zo-1* or claudin. The results demonstrated that high levels of BSFM, potentially due to its indigestible chitin content, may contribute to intestinal damage in crabs, potentially accounting for the higher mortality observed in the BSFM60 group. These findings are consistent with those of Xu et al. (2020), who reported that a BSFM100 diet led to abnormal intestinal development in mirror carp (*Cyprinus carpio* var. specularis). Similarly, several studies have shown that excessive dietary chitin can impair nutrient digestion and absorption, adversely affecting gut health and overall animal welfare (Kroeckel et al., 2012; Yu et al., 2023). Li et al. (2017) also observed increased microvillus fragmentation in the intestines of fish fed BSFM75 and BSFM100 diets. In addition to chitin, protein nutrition has also been shown to influence intestinal morphology and integrity (Hu et al., 2023). Concurrently, several studies have indicated that chitin can serve as a substrate for bacteria that produce chitinase (Huyben et al., 2019). Dietary inclusion of BSFM has been shown to significantly alter the gut microbiota in rainbow trout (*Oncorhynchus mykiss*) (Bruni et al., 2018) and Atlantic cod (*Gadus morhua*) (Zhou et al., 2013). However, a limitation of this study is the absence of direct measurements of chitin content and nutrient digestibility in the experimental diets. Therefore, building upon these findings, future research should prioritize elucidating the specific mechanisms by which high-level BSFM substitution impacts gut health in crustaceans. A key focus should be the systematic analysis of the quantitative relationships between dietary chitin levels and subsequent changes in the gut microbiota structure, intestinal barrier integrity, and systemic inflammatory responses in mud crabs. This line of investigation is crucial to clarify the application limits of BSFM in crustacean diets.

The antioxidant system, which plays a key role in sustaining organismal health, includes enzymes such as SOD, CAT, and GSH-Px, that neutralize reactive oxygen species (ROS) and alleviate oxidative stress (Zhu et al., 2021). In the present investigation, moderate inclusion of BSFM significantly elevated activities of CAT and GSH, and up-regulated the expression of antioxidant-related genes (*trx* and *prx*) in the hepatopancreas, suggesting enhanced antioxidant capacity. Oxidative stress can damage biological macromolecules through lipid peroxidation and protein oxidation. MDA is a well-established marker of lipid peroxidation and nucleic acid degradation, while PC content is commonly used to assess ROS-induced protein oxidation (Guler et al., 2009). In the present study, no significant differences were observed in MDA and PC contents across the dietary treatments. Moreover, the MDA content exhibited a decreasing trend, which aligns with previous findings in mud crabs (Yang et al., 2025) and swimming crabs (Li et al., 2023b). This might be due to the moderate replacement of FM with BSFM, attenuating hepatopancreatic oxidative damage and enhancing the overall antioxidant capacity (Li et al., 2023a). This protective effect can be attributed to an adaptive up-regulation of the antioxidant system, which appears to have counterbalanced potential oxidative insults, thereby safeguarding cellular macromolecules from peroxidation and oxidation. A potential underlying mechanism for this enhanced resilience may be an increase in mitochondrial abundance (Yang et al., 2025). As mitochondria are rich in antioxidant activities and crucial for ROS elimination and redox homeostasis, an increase in their number could significantly bolster the cell's antioxidant defenses. Future research should prioritize elucidating the exact bioactive compounds in BSFM responsible for this effect and the specific signaling pathways involved, to fully exploit BSFM as a functional feed ingredient for promoting crustacean health. In summary, a moderate replacement of FM with BSFM seems to promote hepatopancreatic health not merely by suppressing oxidative stress, but by priming the antioxidant system, enabling the organism to better manage ROS challenges without escalating to biomolecular damage.

### 4.5 Flesh quality and sensory attributes

This study demonstrates that dietary inclusion of BSFM significantly enhances the physical quality of mud crab muscle. Flesh physical characteristics, including textural attributes and liquid-holding capacity, are key indicators of flesh quality and significantly influence consumer acceptance (Xu et al., 2022). As flesh texture is closely linked to diet, firmer textures are generally preferred by consumers (Valente et al., 2013). In this study, BSFM inclusion enhanced the liquid-holding capacity, hardness, springiness, chewiness, and gumminess of mud crab muscle, consistent with findings in grass carp (Hu et al., 2023). Muscle crude lipid content did not differ significantly among dietary groups. While high lipid levels may contribute to softer flesh, texture is also affected by variables such as collagen content and muscle fiber structure (Periago et al., 2005). Myofiber diameter and density are key factors influencing muscle texture (Li et al., 2023c). During this investigation, improved muscle texture may be linked to increased myofiber diameter and decreased fiber density. However, previous studies have reported a positive correlation between muscle texture and fiber density (Li et al., 2023c; Luo et al., 2021; Song et al., 2022). Muscle development involves two main processes: hypertrophy, referring to the enlargement of existing fibers from late embryonic stages to adulthood; and hyperplasia, the formation of new fibers (Valente et al., 2013). Both processes affect muscle cellularity and texture (Li et al., 2022). The firmer muscle texture observed in this study may result from muscle fiber hypertrophy rather than increased fiber density, though the underlying mechanisms require further investigation. Collagen content is another key determinant of flesh texture, with higher levels generally associated with increased firmness (Johnston, 2008). In this study, adding BSFM to the diet increased muscle collagen content, which might explain the improvement in fluid-holding capacity and hardness. Muscle pH is another important quality indicator (Wei et al., 2016), as lower pH can reduce water retention and weaken connective tissue, resulting in softer flesh (Bjørnevik et al., 2017; Lu et al., 2021). The research found that BSFM had no significant influence on fresh muscle pH. However, after 3 d of storage at 4 °C, the pH in the BSFM60 group was considerably higher than that of the control group, indicating improved pH stability. Overall, BSFM supplementation improved muscle texture and quality, enhancing the product's consumer appeal.

This study demonstrates that high-level substitution (60%) of fish meal with BSFM significantly alters the flavor profile of mud crab muscle, primarily by reducing umami intensity and introducing undesirable bitter notes. Taste is a critical sensory attribute that strongly influences consumer acceptance and shapes consumption patterns of aquatic products (Bu et al., 2022). Among the key contributors to the flavor of crab muscle are FAAs and nucleotides, which are recognized as primary taste-active compounds (Luo et al., 2021). Nucleotides and their derivatives not only serve as essential energy sources but also play a pivotal role in enhancing the palatability of aquatic foods. Specifically, ATP catabolism yields AMP and IMP, both of which synergize with FAAs to significantly intensify the umami and sweetness characteristics of crab flesh (Yin et al., 2023). The FAAs typically contribute to umami, sour, bitter, or sweet tastes. Although some FAAs are present in high concentrations in crab flesh, their contribution to flavor may be limited due to their high taste threshold values. The FAAs are generally derived from proteolysis or specific amino acid metabolic pathways. While proteolysis increases the overall FAA concentration, metabolic processes may reduce levels of certain amino acids (Yue et al., 2016). The impact of FAAs on taste perception depends on both their content and taste threshold. To quantitatively assess their contribution, the TAV serves as a useful indicator; a TAV greater than 1 is typically considered to exert a direct influence on flavor (Chen and Zhang, 2007). In this study, nine FAAs with TAVs greater than 1, namely glutamic acid, glycine, alanine, proline, valine, histidine, arginine, methionine, and lysine, were identified as potential contributors to the overall flavor of mud crab muscle. Although the inclusion of BSFM did not significantly alter the TAVs of the umami-related FAAs (aspartic acid and glutamic acid), it notably decreased their absolute concentrations in the muscle tissue. Importantly, a synergistic interaction exists between umami FAAs (aspartic acid and glutamic acid) and flavor-enhancing nucleotides (GMP, IMP, and AMP), collectively enhancing umami perception, as reflected in the EUC values. The EUC reflects the umami intensity, expressed as the amount of monosodium glutamate (MSG) equivalent to that found in 100 g of fresh mud crab muscle (Yin et al., 2023). Umami is an important aspect in determining the overall flavor of aquatic items and contributes significantly to mud crabs' commercial appeal. In this investigation, the EUC value was considerably lower in the BSFM60 group, showing that a high amount of BSFM replacement for FM lowered the umami intensity of crab muscles. This observation is consistent with the results obtained from the electronic tongue analysis. Similar results have been found in investigations on rainbow trout (Mancini et al., 2018). Hypoxanthine, a downstream product of ATP catabolism, is known to enhance the bitterness of aquatic products (Zhang et al., 2021). In the current investigation, BSFM supplementation had no significant effect on the levels of bitter-tasting FAAs (TAV >1) in crab muscle, but it did significantly increase the Hx content, indicating that BSFM may impart an undesirable bitter flavor to the muscle. This observation aligns with the electronic tongue analysis. Moreover, a previous study has shown that arginine, a widely distributed amino acid in seafood, contributes to a more favorable flavor profile that is generally preferred over bitterness by consumers (Chen and Zhang, 2007). This experiment showed a declining trend in arginine content across groups, although the change was not statistically significant. Interestingly, BSFM inclusion significantly increased crab muscle glycine and alanine levels. In contrast, electronic tongue analysis showed a decrease in perceived sweetness. The TAVs capture the taste of individual compounds but do not mask or synergistic interactions between flavor substances (Yu et al., 2024). High Hx levels may have obscured the sweetness, reducing its sensory perception. Bitterness can hide sweetness, according to a previous study (Fan et al., 2022). Electronic tongue analysis showed that BSFM supplementation made crab muscle sourer and less rich and salty. The samples had lower sourness and saltiness than the control solution, as shown by their negative values. This suggests that sourness and saltiness contributed little to muscle flavor. The PCA results showed that the BSFM40 and BSFM60 groups were positioned farther from the control group, suggesting more distinct flavor profiles compared to the BSFM0 group. The loading plot indicated that proximity to PC3 was associated with increased bitterness and sourness, and decreased umami. Consistent with this, the BSFM40 and BSFM60 samples were located closer to PC3 along the horizontal axis than the control group, implying that higher BSFM inclusion levels may negatively impact the overall flavor of mud crab muscle by enhancing undesirable tastes and reducing pleasant ones. However, a limitation of this study is the absence of sensory evaluation to validate these findings. Furthermore, the underlying mechanisms by which BSFM alters muscle flavor in mud crab warrant further investigation.

## 5 Conclusions

In conclusion, the present study demonstrated that BSFM is a promising alternative protein source for partially replacing FM in mud crab diets. Substitution levels up to 30% had no negative effects on growth performance, amino acid composition, and fatty acid profiles of the hepatopancreas and muscle, while also improving hepatopancreas and intestinal health and enhancing muscle texture. However, higher inclusion levels (40% and 60%) did not impair growth performance but reduced nutritional value and altered muscle flavor. Notably, the 60% replacement level induced intestinal damage and increased mortality. Therefore, BSFM inclusion should not exceed 30% (122.5 g/kg) in mud crab diets.

## Credit Author Statement

**Tiantian Xu:** Writing – original draft, Data curation, Conceptualization. **Yuhang Yang:** Validation, Data curation. **Xiaoyi Zhao:** Software, Formal analysis. **Shichao Xie:** Project administration, Investigation. **Yinqiu Tian:** Data curation. **Tingting Zhu:** Resources, Methodology. **Min Jin:** Funding acquisition, Formal analysis. **Qicun Zhou:** Writing – review & editing, Supervision, Project administration, Methodology, Funding acquisition.

## Declaration of competing interest

We declare that we have no financial and personal relationships with other people or organizations that can inappropriately influence our work, and there is no professional or other personal interest of any nature or kind in any product, service and/or company that could be construed as influencing the content of this paper.

## References

[bib1] Alagappan S., Rowland D., Barwell R., Cozzolino D., Mikkelsen D., Olarte Mantilla S.M. (2022). Organic side streams (bioproducts) as substrate for black soldier fly (*Hermetia illucens*) intended as animal feed: chemical safety issues. Anim Prod Sci.

[bib2] Albayati A.I., Attee R.S., Al-Khshali M.S. (2025). Effect of replacing soybean meal with larvae meal of black soldier fly *Hermetia illucens* inagrowth performance and some biochemical blood parameters of common carp *Cyprinus carpio* L. Iraqi J Agric Sci.

[bib3] Anedda R., Melis R., Palomba A., Vitangeli I., Biosa G., Braca A. (2023). Balanced replacement of fish meal with *Hermetia illucens* meal allows efficient hepatic nutrient metabolism and increases fillet lipid quality in gilthead sea bream (*Sparus aurata*). Aquaculture.

[bib4] AOAC (2006). Official methods of analysis.

[bib5] Belghit I., Liland N.S., Gjesdal P., Biancarosa I., Menchetti E., Li Y. (2019). Black soldier fly larvae meal can replace fish meal in diets of sea-water phase Atlantic salmon (*Salmo salar*). Aquaculture.

[bib6] Bjørnevik M., Hansen H., Roth B., Foss A., Vikingstad E., Solberg C. (2017). Effects of starvation, subsequent feeding and photoperiod on flesh quality in farmed cod (*Gadus morhua*). Aquac Nutr.

[bib7] Bruni L., Pastorelli R., Viti C., Gasco L., Parisi G. (2018). Characterisation of the intestinal microbial communities of rainbow trout (*Oncorhynchus mykiss*) fed with *Hermetia illucens* (black soldier fly) partially defatted larva meal as partial dietary protein source. Aquaculture.

[bib8] Bruni L., Belghit I., Lock E.J., Secci G., Taiti C., Parisi G. (2020). Total replacement of dietary fish meal with black soldier fly (*Hermetia illucens*) larvae does not impair physical, chemical or volatile composition of farmed Atlantic salmon (*Salmo salar L.*). J Sci Food Agric.

[bib9] Bruni L., Randazzo B., Cardinaletti G., Zarantoniello M., Mina F., Secci G. (2020). Dietary inclusion of full-fat *Hermetia illucens* prepupae meal in practical diets for rainbow trout (*Oncorhynchus mykiss*): Lipid metabolism and fillet quality investigations. Aquaculture.

[bib10] Bu X., Song Y., Cai X., Tang L., Huang Q., Wang X. (2022). Enhancement of protein deposition and meat quality of male Chinese mitten crab (*Eriocheir sinensis*): application of myo-inositol in crustacean nutrition. LWT--Food Sci Technol.

[bib11] Charlton A.J., Dickinson M., Wakefield M.E., Fitches E., Kenis M., Han R. (2015). Exploring the chemical safety of fly larvae as a source of protein for animal feed. J Insects Food Feed.

[bib12] Chen D.W., Zhang M. (2007). Non-volatile taste active compounds in the meat of Chinese mitten crab (*Eriocheir sinensis*). Food Chem.

[bib13] China National Standard (2017).

[bib14] China National Standard (2022).

[bib15] Deng Y., Zhan W.H., Xie S.C., Peng H.Y., Cao H.Q., Tang Z. (2025). Multi-omics analysis revealed the effects of different astaxanthin sources on the antioxidant properties of *Scylla paramamosain*. Food Chem.

[bib16] Diener S., Zurbrugg C., Tockner K. (2009). Conversion of organic material by black soldier fly larvae: establishing optimal feeding rates. Waste Manag Res.

[bib17] Eide L.H., Rocha S.D.C., Morales-Lange B., Kuiper R.V., Dale O.B., Djordjevic B. (2024). Black soldier fly larvae (*Hermetia illucens*) meal is a viable protein source for Atlantic salmon (*Salmo salar*) during a large-scale controlled field trial under commercial-like conditions. Aquaculture.

[bib18] Esteban M.A., Cuesta A., Ortuno J., Meseguer J. (2001). Immunomodulatory effects of dietary intake of chitin on gilthead seabream (*Sparus aurata* L.) innate immune system. Fish Shellfish Immunol.

[bib19] European Commission (2006). Commission Regulation (EC) no 1881/2006 of 19 December 2006: setting maximum levels for certain contaminants in foodstuffs. Off J Eur Communities.

[bib20] Fan L., Xiao T., Xian C., Ding W., Wang X. (2022). Effect of short-term frozen storage on taste of gonads of female *Eriocheir sinensis* and the classification of taste quality combined with sensory evaluation and fuzzy logic model. Food Chem.

[bib21] FAO (2022). Brief to the State of world fisheries and aquaculture.

[bib22] Ganesan A.R., Mohan K., Kandasamy S., Surendran R.P., Kumar R., Rajan D.K. (2024). Food waste-derived black soldier fly (*Hermetia illucens*) larval resource recovery: a circular bioeconomy approach. Process Saf Environ.

[bib23] Gaudioso G., Marzorati G., Faccenda F., Weil T., Lunelli F., Cardinaletti G. (2021). Processed animal proteins from insect and poultry by-products in a fish meal-free diet for rainbow trout: impact on intestinal microbiota and inflammatory markers. Int J Mol Sci.

[bib24] Georgescu I.M., Zvoristeanu O.V., Ghita M., Negreanu C.N., Raba D.N., Ciobotaru-Pirvu E. (2020). Monitoring the lead contamination of food products of non-animal origin in different regions from Romania in 2019. Sci Pap Ser D Anim S.

[bib25] Guler G., Turkozer Z., Ozgur E., Seyhan N. (2009). Antioxidants alleviate electric field-induced effects on lung tissue based on assays of heme oxygenase-1, protein carbonyl content, malondialdehyde, nitric oxide, and hydroxyproline. Sci Total Environ.

[bib26] Guo Y.R., Gu S.Q., Wang X.C., Zhuang K.J., Wang S., Shi J. (2014). Nutrients and non-volatile taste compounds in Chinese mitten crab by-products. Fisheries Sci.

[bib27] Hadji R., Urien N., Uher E., Fechner L.C., Lebrun J.D. (2016). Contribution of aqueous and dietary uptakes to lead (Pb) bioaccumulation in *Gammarus pulex*: from multipathway modeling to in situ validation. Ecotoxicol Environ Saf.

[bib28] Henry M.A., Gasco L., Chatzifotis S., Piccolo G. (2018). Does dietary insect meal affect the fish immune system? The case of mealworm, *Tenebrio molitor* on European sea bass, *Dicentrarchus labrax*. Dev Comp Immunol.

[bib29] Holme M., Southgate P.C., Zeng C.S. (2007). Survival, development and growth response of mud crab, *Scylla serrata*, megalopae fed semi-purified diets containing various fish oil: corn oil ratios. Aquaculture.

[bib30] Hu Y., Huang Y., Tang T., Zhong L., Chu W., Dai Z. (2020). Effect of partial black soldier fly (*Hermetia illucens* L.) larvae meal replacement of fish meal in practical diets on the growth, digestive enzyme and related gene expression for rice field eel (*Monopterus albus*). Aquac Rep.

[bib31] Hu Z., Li H., Liu S., Xue R., Sun J., Ji H. (2023). Assessment of black soldier fly (*Hermetia illucens*) larvae meal as a potential substitute for soybean meal on growth performance and flesh quality of grass carp *Ctenopharyngodon idellus*. Anim Nutr.

[bib32] Huang T., Guo B., Zheng J., Li M., Chen Y., Li X. (2024). Combined supplementation of hydroxyproline and vitamin C improved the growth and flesh quality of Pacific white shrimp (*Litopenaeus vanname*) cultured in low salinity water. Aquacult Fish.

[bib33] Huyben D., Vidaković A., Werner Hallgren S., Langeland M. (2019). High-throughput sequencing of gut microbiota in rainbow trout (*Oncorhynchus mykiss*) fed larval and pre-pupae stages of black soldier fly (*Hermetia illucens*). Aquaculture.

[bib34] Jiang B., Sun Y., Li W., Liu C., Wen C., Li A. (2022). Effects of dietary black soldier fly (*Hermetia illucens* Linnaeus) on the disease resistance of juvenile grouper (*Epinephelus coioides*). Fish Shellfish Immunol.

[bib35] Jin M., Luo J.X., Zhu T.T., Fang F., Xie S.C., Lu J.J. (2024). Examination of role of the AMP-activated protein kinase (Ampk) signaling pathway during low salinity adaptation in the mud crab, *Scylla paramamosain*, with reference to glucolipid metabolism. Aquaculture.

[bib36] Johnston I.A., Børresen T. (2008). Improving seafood products for the consumer.

[bib37] Kari Z.A., Kabir M.A., Dawood M.A.O., Razab M.K.A.A., Ariff N.S.N.A., Sarkar T. (2022). Effect of fish meal substitution with fermented soy pulp on growth performance, digestive enzyme, amino acid profile, and immune-related gene expression of African catfish (*Clarias gariepinus*). Aquaculture.

[bib38] Katya K., Borsra M.Z.S., Ganesan D., Kuppusamy G., Herriman M., Salter A. (2017). Efficacy of insect larval meal to replace fish meal in juvenile barramundi, *Lates calcarifer* reared in freshwater. Int Aquacult Res.

[bib39] Keetanon A., Chuchird N., Phansawat P., Kitsanayanyong L., Chou C.C., Verstraete P. (2024). Effects of black soldier fly larval meal on the growth performance, survival, immune responses, and resistance to *Vibrio parahaemolyticus* infection of Pacific white shrimp (*Litopenaeus vannamei*). Aquac Int.

[bib40] Kim K., Park Y., Je H.W., Seong M., Damusaru J.H., Kim S. (2019). Tuna byproducts as a fish-meal in tilapia aquaculture. Ecotoxicol Environ Saf.

[bib41] Kroeckel S., Harjes A.G.E., Roth I., Katz H., Wuertz S., Susenbeth A. (2012). When a turbot catches a fly: evaluation of a pre-pupae meal of the black soldier fly (*Hermetia illucens*) as fish meal substitute-growth performance and chitin degradation in juvenile turbot (*Psetta maxima*). Aquaculture.

[bib42] Lanes C.F.C., Pedron F.A., Bergamin G.T., Bitencourt A.L., Dorneles B.E.R., Villanova J.C.V. (2021). Black soldier fly (*Hermetia illucens*) larvae and prepupae defatted meals in diets for zebrafish (*Danio rerio*). Animals.

[bib43] Li S., Ji H., Zhang B., Zhou J., Yu H. (2017). Defatted black soldier fly (*Hermetia illucens*) larvae meal in diets for juvenile Jian carp (*Cyprinus carpio var.* Jian): growth performance, antioxidant enzyme activities, digestive enzyme activities, intestine and hepatopancreas histological structure. Aquaculture.

[bib44] Li L., Zhang Y., Wang J., Lu S., Cao Y., Tang C. (2020). History traces of HCHs and DDTs by groundwater dating and their behaviours and ecological risk in northeast China. Chemosphere.

[bib45] Li H., Hu Z., Liu S., Sun J., Ji H. (2022). Influence of dietary soybean meal replacement with yellow mealworm (*Tenebrio molitor*) on growth performance, antioxidant capacity, skin color, and flesh quality of mirror carp (*Cyprinus carpio* var. specularis). Aquaculture.

[bib47] Li Z., Han C., Wang Z., Li Z., Ruan L., Lin H. (2023). Black soldier fly pulp in the diet of golden pompano: effect on growth performance, liver antioxidant and intestinal health. Fish Shellfish Immunol.

[bib48] Li X.K., Li P., Zhou Q.C., Yang Y.H., Xie S.C., Guo C. (2023). Application prospect of replacement of fish meal with spray-dried egg meal in diets for swimming crab (*Portunus trituberculatus*). Aquac Rep.

[bib46] Li H., Xue R., Sun J., Ji H. (2023). Improving flesh quality of grass carp (*Ctenopharyngodon idellus*) by completely replacing dietary soybean meal with yellow mealworm (*Tenebrio molitor*). Anim Nutr.

[bib49] Liu Z.H., Xie W.W., Zan G.X., Gao C.Q., Yan H.C., Zhou J.Y. (2021). Lauric acid alleviates deoxynivalenol-induced intestinal stem cell damage by potentiating the Akt/mTORC1/S6K1 signaling axis. Chem Biol Interact.

[bib50] Livak K.J., Schmittgen T.D. (2001). Analysis of relative gene expression data using real-time quantitative PCR and the 2^−ΔΔCt^ method. Methods.

[bib51] Long X., Yang W., Gu Y., Xiao P., Yang Y., Deng J. (2024). The American cockroach (*Periplaneta americana*) residue could partially replace the dietary fish meal in the juvenile Nile tilapia (*Oreochromis niloticus*). Aquac Rep.

[bib52] Lu H.B., Ma Y.Y., Hu C.T., Lin Q.Y., Yue J.J., Chen L.Q. (2021). The individual and combined effects of hypoxia and high-fat diet feeding on nutrient composition and flesh quality in Nile tilapia (*Oreochromis niloticus*). Food Chem.

[bib53] Luo J.X., Zhu T.T., Jin M., Cheng X., Yuan Y., Wang X.X. (2020). Influence of dietary zinc on growth, zinc bioaccumulation and expression of genes involved in antioxidant and innate immune in juvenile mud crabs (*Scylla paramamosain*). Br J Nutr.

[bib54] Luo J.X., Monroig O., Zhou Q.C., Tocher D.R., Yuan Y., Zhu T.T. (2021). Environmental salinity and dietary lipid nutrition strategy: effects on flesh quality of the marine euryhaline crab *Scylla paramamosain*. Food Chem.

[bib55] Makkar H.P.S., Tran G., Heuzé V., Ankers P. (2014). State-of-the-art on use of insects as animal feed. Anim Feed Sci Technol.

[bib56] Mancini S., Medina I., Iaconisi V., Gai F., Basto A., Parisi G. (2018). Impact of black soldier fly larvae meal on the chemical and nutritional characteristics of rainbow trout fillets. Animal.

[bib57] Mikolajczak Z., Rawski M., Mazurkiewicz J., Kieronczyk B., Kolodziejski P., Pruszynska-Oszmalek E. (2022). The first insight into black soldier fly meal in brown trout nutrition as an environmentally sustainable fish meal replacement. Animal.

[bib58] Mohanty B.P., Mahanty A., Ganguly S., Mitra T., Karunakaran D., Anandan R. (2019). Nutritional composition of food fishes and their importance in providing food and nutritional security. Food Chem.

[bib59] Monteiro dos Santos D.K., Santana T.M., de Matos Dantas F., Farias ABdS., Epifânio C.M.F., Prestes A.G. (2022). Defatted black soldier fly larvae meal as a dietary ingredient for tambaqui (*Colossoma macropomum*): digestibility, growth performance, haematological parameters, and carcass composition. Aquac Res.

[bib60] Monteiro dos Santos D.K., Rodrigues de Freitas O., Oishi C.A., Leao da Fonseca F.A., Parisi G., Uribe Goncalves L. (2023). Full-fat black soldier fly larvae meal in diet for Tambaqui, *Colossoma macropomum*: digestibility, growth performance and economic analysis of feeds. Animals.

[bib61] Navajas-Porras B., Delgado-Osorio A., Hinojosa-Nogueira D., Pastoriza S., Almécija-Rodríguez M.C., Rufián-Henares J. (2024). Improved nutritional and antioxidant properties of black soldier fly larvae reared on spent coffee grounds and blood meal by-products. Food Res Int.

[bib62] Naylor R.L., Hardy R.W., Buschmann A.H., Bush S.R., Cao L., Klinger D.H. (2021). A 20-year retrospective review of global aquaculture. Nature.

[bib63] Panini R.L., Pinto S.S., Nóbrega R.O., Vieira F.F., Fracalossi D.M., Samuels R.I. (2017). Effects of dietary replacement of fishmeal by mealworm meal on muscle quality of farmed shrimp *Litopenaeus vannamei*. Food Res Int.

[bib64] Peng H., Jin M., Cui X., Cao H., Zhan W., Deng Y. (2025). Dietary butyrate promoted nutrient deposition by increasing carbohydrate utilization and energy supply in *Scylla paramamosain*. Aquaculture.

[bib65] Periago M.J., Ayala M.D., López-Albors O., Abdel I., Martínez C., García-Alcázar A. (2005). Muscle cellularity and flesh quality of wild and farmed sea bass, *Dicentrarchus labrax* L. Aquaculture.

[bib66] Picard B., Lefèvre F., Lebret B. (2012). Meat and fish flesh quality improvement with proteomic applications. Anim Front.

[bib67] Pulido L., Secci G., Maricchiolo G., Gasco L., Gai F., Serra A. (2022). Effect of dietary black soldier fly larvae meal on fatty acid composition of lipids and sn-2 position of triglycerides of marketable size gilthead sea bream fillets. Aquaculture.

[bib68] Purschke B., Scheibelberger R., Axmann S., Adler A., Jager H. (2017). Impact of substrate contamination with mycotoxins, heavy metals and pesticides on the growth performance and composition of black soldier fly larvae (*Hermetia illucens*) for use in the feed and food value chain. Food Addit Contam.

[bib69] Qian Y., Zheng M.H., Zhang B., Gao L.R., Liu W.B. (2006). Determination and assessment of HCHs and DDTs residues in sediments from Lake Dongting, China. Environ Monit Assess.

[bib70] Rodríguez-Añón J.A., Proupin-Castineiras J. (2005). Thermal analysis. Fundamentals and applications to material characterization.

[bib71] Rørå A., Regost C., Lampe J. (2003). Liquid holding capacity, texture and fatty acid profile of smoked fillets of Atlantic salmon fed diets containing fish oil or soybean oil. Food Res Int.

[bib72] Sangiacomo C., Trombetta L., Susini F., Brogi L., Licitra R., Machese M. (2025). *Hermetia illucens* meal from different substrates for replacing fishmeal: study on zebrafish as fish model. Aquac Rep.

[bib73] Siddaiah G.M., Kumar R., Kumari R., Chandan N.K., Debbarma J., Damle D.K. (2023). Dietary fishmeal replacement with *Hermetia illucens* (Black soldier fly, BSF) larvae meal affected production performance, whole body composition, antioxidant status, and health of snakehead (*Channa striata*) juveniles. Anim Feed Sci Technol.

[bib74] Song D.Y., Yun Y.H., He Z.J., Mi J.L., Wang L.M., Jin M. (2022). Fillet texture, physicochemical indexes, muscle cellularity and molecular expression in muscle of Yellow River carp (*Cyprinus carpio haematopterus*) in response to dietary hydroxyproline supplementation. Aquaculture.

[bib75] Spranghers T., Ottoboni M., Klootwijk C., Ovyn A., Deboosere S., De Meulenaer B. (2017). Nutritional composition of black soldier fly (*Hermetia illucens*) prepupae reared on different organic waste substrates. J Sci Food Agric.

[bib76] Stejskal V., Tran H.Q., Prokesova M., Zare M., Gebauer T., Policar T. (2023). Defatted black soldier fly (*Hermetia illucens*) in pikeperch (*Sander lucioperca*) diets: effects on growth performance, nutrient digestibility, fillet quality, economic and environmental sustainability. Anim Nutr.

[bib77] Takakuwa F., Tanabe R., Nomura S., Inui T., Yamada S., Biswas A. (2022). Availability of black soldier fly meal as an alternative protein source to fish meal in red sea bream (*Pagrus major*, Temminck & Schlegel) fingerling diets. Aquac Res.

[bib78] Tang M., Qu Z., Shi W., Wang X., Wu X. (2024). A comparative study on the flavour of wild Chinese mitten crab (*Eriocheir sinensis*) along the eastern coast of China. J Food Compos Anal.

[bib79] Tang Z., Xie S., Cui Y., Zhan W., Deng Y., Peng H. (2024). Vitamin C as a functional enhancer in the non-specific immune defense, antioxidant capacity and resistance to low-temperature stress of juvenile mud crab, *Scylla paramamosain*. Fish Shellfish Immunol.

[bib80] Tippayadara N., Dawood M.A.O., Krutmuang P., Hoseinifar S.H., Doan H.V., Paolucci M. (2021). Replacement of fish meal by black soldier fly (*Hermetia illucens*) larvae meal: effects on growth, haematology, and skin mucus immunity of Nile tilapia, *Oreochromis niloticus*. Animals.

[bib81] Tocher D.R., Betancor M.B., Sprague M., Olsen R.E., Napier J.A. (2019). Omega-3 long-chain polyunsaturated fatty acids, EPA and DHA: bridging the gap between supply and demand. Nutrients.

[bib82] Tran H.Q., von Siebenthal E.W., Luce J.B., Nguyen T.T., Tomčala A., Stejskal V. (2024). Complementarity of insect meal and poultry by-product meal as replacement for fishmeal can sustain the production performance of European perch (*Perca fluviatilis*), reduce economic fish-in fish-out ratio and food-feed competition, and influence the environmental indices. Aquaculture.

[bib83] Valente L.M.P., Moutou K.A., Conceição L.E.C., Engrola S., Fernandes J.M.O., Johnston I.A. (2013). What determines growth potential and juvenile quality of farmed fish species?. Rev Aquacult.

[bib84] Villanueva-Gutiérrez E., Rodriguez-Armenta C., González-Félix M.L., Perez-Velazquez M. (2022). Incorporating hydrolyzed soy protein or black soldier fly (*Hermetia illucens*) larvae meal into feeds for *Totoaba macdonaldi*. Aquaculture.

[bib85] Wang X.X., Liu J.J., Cui S.H., Wang Z.Y., Ye Z.H., Xu Y.F. (2024). Effects of terrestrial animal fats replacing dietary fish oil on the growth performance, antioxidant capacity and lipid metabolism in juvenile *Scylla paramamosain*. Aquac Rep.

[bib86] Wei Z., Ma J., Pan X., Mu H., Li J., Shentu J. (2016). Dietary hydroxyproline improves the growth and muscle quality of large yellow croaker *Larimichthys crocea*. Aquaculture.

[bib87] Xiao X., Jin P., Zheng L., Cai M., Yu Z., Yu J. (2018). Effects of black soldier fly (*Hermetia illucens*) larvae meal protein as a fishmeal replacement on the growth and immune index of yellow catfish (*Pelteobagrus fulvidraco*). Aquac Res.

[bib88] Xiong J., Jin M., Yuan Y., Luo J.X., Lu Y., Zhou Q.C. (2018). Dietary nucleotide-rich yeast supplementation improves growth, innate immunity and intestinal morphology of Pacific white shrimp (*Litopenaeus vannamei*). Aquac Nutr.

[bib89] Xu X., Ji H., Yu H., Zhou J. (2020). Influence of dietary black soldier fly (*Hermetia illucens* Linnaeus) pulp on growth performance, antioxidant capacity and intestinal health of juvenile mirror carp (*Cyprinus carpio* var. specularis). Aquac Nutr.

[bib90] Xu X., Yang H., Zhang C., Bian Y., Yao W., Xu Z. (2022). Effects of replacing fishmeal with cottonseed protein concentrate on growth performance, flesh quality and gossypol deposition of largemouth bass (*Micropterus salmoides*). Aquaculture.

[bib91] Xu T.T., Yang Z., Xie S.C., Zhu T.T., Zhao W.L., Jin M. (2024). Evaluation of cottonseed oil as a substitute for fish oil in the commercial diet for juvenile swimming crab (*Portunus trituberculatus*). Anim. Nutr.

[bib92] Yang Q.B., Fan R., Ma Z.H., Jiang S., Huang J.H., Yang L.S. (2023). Effects of replacing dietary fishmeal with zymolytic black soldier fly larvae on the growth performance of the mud crab (*Scylla paramamosain*) larvae. Isr J Aquacult Bamid.

[bib111] Yang Y., Jin M., Li X., Xie S.C., Guo C., Zhang X. (2024). Evaluation of spray-dried blood meal for application in commercial-like feed for juvenile swimming crab (*Portunus trituberculatus*). Aquac Rep.

[bib93] Yang Y., Zhu T., Jin M., Li X., Xie S., Cui Y. (2025). Black soldier fly larvae oil can partially replace fish oil in the diet of the juvenile mud crab (*Scylla paramamosain*). Anim Nutr.

[bib94] Yin M., Xi Y., Shi Y., Qiu Z., Matsuoka R., Wang H. (2023). Effects of temperature fluctuations on non-volatile taste compounds in tilapia fillets (*Oreochromis niloticus*). Food Chem.

[bib95] Yu Z., Sun Z., Ou B., Zhou M., Huang Y., Tan X. (2023). Effects of partial replacement of fish meal with black soldier fly (*Hermetia illucens*) larvae meal on growth performance, lipid metabolism and hepatointestinal health of juvenile golden pompano (*Trachinotus ovatus*). Aquac Rep.

[bib96] Yu K.J., Zhu G.F., Shi C., Ye Y.F., Li R.H., Mu C.K. (2024). Overwintering temperature affects the nutrient composition and non-volatile flavor substances of female adult mud crab *Scylla paramamosain* in recirculating aquaculture systems (RASs). Aquaculture.

[bib97] Yuan Y., Wang X.X., Jin M., Sun P., Zhou Q.C. (2019). Influence of different lipid sources on growth performance, oxidation resistance and fatty acid profiles of juvenile swimming crab, *Portunus trituberculatus*. Aquaculture.

[bib98] Yuan Y., Wang X.X., Jin M., Jiao L.F., Sun P., Betancor M.B. (2020). Modification of nutritional values and flavor qualities of muscle of swimming crab (*Portunus trituberculatus*): application of a dietary lipid nutrition strategy. Food Chem.

[bib99] Yue J., Zhang Y., Jin Y., Deng Y., Zhao Y. (2016). Impact of high hydrostatic pressure on non-volatile and volatile compounds of squid muscles. Food Chem.

[bib100] Zarantoniello M., de Oliveira A.A., Sahin T., Freddi L., Torregiani M., Tucciarone I. (2023). Enhancing rearing of European seabass (*Dicentrarchus labrax*) in aquaponic systems: investigating the effects of enriched black soldier fly (*Hermetia illucens*) prepupae meal on fish welfare and quality traits. Animals.

[bib101] Zaretabar A., Ouraji H., Abedian Kenari A., Yeganeh S., Esmaeili N., Keramat Amirkolaee A. (2021). One step toward aquaculture sustainability of a carnivorous species: fish meal replacement with barley protein concentrate plus wheat gluten meal in Caspian brown trout (*Salmo trutta* caspius). Aquac Rep.

[bib102] Zeng Q.L., Dong G.Q., Tian L.L., Wu H., Ren Y.J., Tamir G. (2020). High altitude is beneficial for antioxidant components and sweetness accumulation of rabbiteye blueberry. Front Plant Sci.

[bib103] Zhang L., Yin M., Zheng Y., Xu C.H., Tao N.P., Wu X. (2021). Brackish water improves the taste quality in meat of adult male *Eriocheir sinensis* during the postharvest temporary rearing. Food Chem.

[bib104] Zhang Y., Zhou X.Q., Jiang W.D., Wu P., Liu Y., Ren H.M. (2024). Emerging role of vitamin D_3_ in alleviating intestinal structure injury caused by *Aeromonas hydrophila* in grass carp (*Ctenopharyngodon idella*). Anim Nutr.

[bib105] Zhang Y., Gu Y., Tian Y., Zhan W., Deng Y., Xie S. (2025). Evaluation of *Clostridium autoethanogenum* protein as a fish meal substitute in diets for juvenile mud crab (*Scylla paramamosain*). Aquac Rep.

[bib106] Zheng P., Han T., Li X., Wang J., Su H., Xu H. (2020). Dietary protein requirement of juvenile mud crab *Scylla paramamosain*. Aquaculture.

[bib107] Zhou Z., Karlsen O., He S., Olsen R.E., Yao B., Ringo E. (2013). The effect of dietary chitin on the autochthonous gut bacteria of Atlantic cod (*Gadus morhua* L.). Aquac Res.

[bib108] Zhu S., Long X., Turchini G.M., Deng D., Cheng Y., Wu X. (2021). Towards defining optimal dietary protein levels for male and female sub-adult Chinese mitten crab, *Eriocheir sinensis* reared in earthen ponds: performances, nutrient composition and metabolism, antioxidant capacity and immunity. Aquaculture.

[bib109] Zhu Y.W., Zhou X.R., Chen Y.P., Liu Z.Y., Jiang S., Chen G.L. (2022). Exploring the relationships between perceived umami intensity, umami components and electronic tongue responses in food matrices. Food Chem.

[bib110] Zhuang K.J., Wu N., Wang X.C., Wu X.G., Wang S., Long X. (2016). Effects of 3 feeding modes on the volatile and nonvolatile compounds in the edible tissues of female Chinese mitten crab (*Eriocheir sinensis*). J Food Sci.

