# Supplementary material for: Black soldier fly (Hermetia illucens) larva meal as a sustainable protein enhances growth, health and flesh quality of Scyllaparamamosain
Source: Anim Nutr. 2026 Jun 8;26:446–66. doi: 10.1016/j.aninu.2025.11.016 (PMC13351848; doi:10.1016/j.aninu.2025.11.016)
Supplement: Multimedia component 1 [file mmc1.docx]

**Table S1** Abbreviations and full names of genes.

| Abbreviations | Full names |
| --- | --- |
| *cu/zn-sod* | Copper/zinc superoxide dismutase |
| *il16* | Interleukin 16 |
| *litaf* | Lipopolysaccharide induced TNF factor |
| *mlck* | Myosin light chain kinase |
| *prx* | Thioredoxin peroxidase |
| *rab6a* | Ras-related protein Rab-6A |
| *tgf* | Transforming growth factor beta regulator 1-like protein |
| *toll1* | Toll-like receptor 1 |
| *toll2* | Toll-like receptor 2 |
| *trx* | Thioredoxin |
| *zo-1* | Tight junction protein ZO-1 |

**Table S2** Sodium citrate buffer system compositions for total amino acid analysis.

| Items | pH |  |  |  |  |
| --- | --- | --- | --- | --- | --- |
|  | 1 | 2 | 3 | 4 | 5 |
| The concentration of sodium, N | 0.16 | 0.20 | 0.20 | 1.20 | 0.20 |
| Density, g/cm^3^ | 1.02 | 1.02 | 1.02 | 1.06 | 1.00 |
| pH | 3.30 | 3.20 | 4.00 | 4.90 | - |
| Distillated water, mL | 700.00 | 700.00 | 700.00 | 700.00 | 700.00 |
| Trisodium citrate dehydrate, g | 6.19 | 7.74 | 13.31 | 26.67 | - |
| Sodium hydroxide, g | - | - | - | - | 8.00 |
| Sodium chloride, g | 5.66 | 7.07 | 3.74 | 54.35 | - |
| Citric acid monohydrate, g | 19.80 | 22.00 | 12.80 | 6.10 | - |
| Alcohol, mL | 130.00 | 20.00 | 4.00 | - | 100.00 |
| Benzyl alcohol, mL | - | - | - | 5.00 | - |
| Dihydroxyethyl sulfide, mL | 5.00 | 5.00 | 5.00 | - | - |
| Polyethylen, mL | 4.00 | 4.00 | 4.00 | 4.00 | 4.00 |
| Octanoic acid, mL | 0.10 | 0.10 | 0.10 | 0.10 | 0.10 |

- = indicates could not be determined.

**Table S3** Linear gradient elution of high-performance liquid chromatography in determination of free amino acids.

| Time, min | A, % | B, % | Flow rate, mL/min |
| --- | --- | --- | --- |
| 0.0 | 92 | 8 | 1.0 |
| 27.5 | 40 | 60 | 1.0 |
| 31.5 | 0 | 100 | 1.5 |
| 32.0 | 0 | 100 | 1.5 |
| 34.0 | 0 | 100 | 1.0 |
| 35.5 | 92 | 8 | 1.0 |

**Table S4** Primers for real-time quantitative PCR.

| Genes | Nucleotide sequence (5’-3’) | Primer efficiency, % | GenBank No. or references |
| --- | --- | --- | --- |
| *trx* | F: AGGAAGACTTCAGGAACCGG | 99.15 | JQ863320.1 |
|  | R: CGAACTTGTCCACCACCTTG |  |  |
| *prx* | F: TGGGGACAAGATTCGTATG | 108.9 | JX133230.1 |
|  | R: AGCAAGGGAGCAAGACAGA |  |  |
| *cu/zn-sod* | F: ATCACCCCAACCTCAACAA | 106.3 | FJ774661 |
|  | R: ATCATCCACAACTCCCCAC |  |  |
| *litaf* | F: GACCAGTTCTCCCAATCCCA | 93.87 | MK510015.1 |
|  | R: AGCCTGCAAAGGTGTGAATG |  |  |
| *rab6a* | F: CTTCCACCAGACCTCCAAGT | 98.44 | Base on transcriptome |
|  | R: ACATTGTATCCCGCCTTTGC |  | (PRJNA634782) |
| *il-16* | F: TGGCAGAGGTTACAGGTCACGGTTAT | 91.75 | Gu et al., 2019 |
|  | R: GGAGTCTGGTGTTCGTCACTGTTTCT |  |  |
| *tgf* | F: CTTTGTGTTGGGCAGTGGAA | 90.18 | Base on transcriptome |
|  | R: CTGCTCCCACTCTTTCTTGC |  | (PRJNA634782) |
| *toll1* | F: TCTTAGCAGCAATAGAATCCGCA | 97.78 | Wei et al., 2024 |
|  | R: TCCAAGGAGCATCACTTGGATCA |  |  |
| *toll2* | F: GTCCTCTCGTCCAGCTTCATTGAGA | 97.69 | Wei et al., 2024 |
|  | R: CAGGTGGGATCTTGCCATAGACAAT |  |  |
| *zo-1* | F: GTACTGGACATCACCCCCAA | 98.57 | Base on transcriptome |
|  | R: AGCTTCTCCAGCTTCACACT |  | (PRJNA634782) |
| claudin | F: GTACTGGACATCACCCCCAA | 90.52 | Base on transcriptome |
|  | R: AGCTTCTCCAGCTTCACACT |  | (PRJNA634782) |
| *mlck* | F: CCCAACCCAACCAAACCAAA | 95.53 | Base on transcriptome |
|  | R: GATTGGGCTGGGTTGAGTTG |  | (PRJNA634782) |
| β-Actin | F: GCCCTTCCTCACGCTATCCT | 102.2 | KC795683.1 |
|  | R: GCGGCAGTGGTCATCTCCT |  |  |
|  | R: AGGTCGCAAACTTCTTCTTTG |  |  |

**Table S5** High-performance liquid chromatography (HPLC) gradient eluent compositions and program for free nucleotide analysis (%)^1^.

| Time, min | Mobile phase A | Mobile phase B |
| --- | --- | --- |
| 0 | 100 | 0 |
| 5 | 100 | 0 |
| 15 | 90 | 10 |
| 25 | 0 | 100 |
| 30 | 0 | 100 |
| 35 | 100 | 0 |
| 40 | 100 | 0 |

^1^The HPLC parameters were as follows: a Diamonsil C18 (4.6 mm × 250 mm) liquid chroma tography column; a column temperature of 30 °C; a flow rate of 0.8 mL/min; an injection volume of 5 μL; and a UV detector wavelength of 254 nm. Mobile phase A: methanol/water/phosphorous acid = 50/950/0.5 mL; Mobile phase B: methanol/water/phosphorous acid = 800/200/0.5 mL.

**Table S6** Amino acid compositions (g/100 g) of hepatopancreas of mud crabs fed with different experimental diets for 8 weeks (wet basis).

| Amino acids | Replacement of FM with BSFM, % | | | | | | SEM | *P*-value | | |
| --- | --- | --- | --- | --- | --- | --- | --- | --- | --- | --- |
|  | 0 | 10 | 20 | 30 | 40 | 60 |  | ANOVA | Linear | Quadratic |
| **EAA** |  |  |  |  |  |  |  |  |  |  |
| Arginine | 0.64^a^ | 0.59^ab^ | 0.55^ab^ | 0.55^ab^ | 0.52^b^ | 0.52^b^ | 0.013 | 0.010 | <0.001 | 0.136 |
| Histidine | 0.31^ab^ | 0.29^abc^ | 0.29^abc^ | 0.32^a^ | 0.25^bc^ | 0.24^b^ | 0.008 | 0.004 | 0.001 | 0.059 |
| Isoleucine | 0.46^ab^ | 0.43^ab^ | 0.39^ab^ | 0.51^a^ | 0.39^ab^ | 0.38^b^ | 0.014 | 0.025 | 0.068 | 0354 |
| Leucine | 0.78^ab^ | 0.77^ab^ | 0.71^b^ | 0.85^a^ | 0.69^b^ | 0.67^b^ | 0.017 | 0.002 | 0.007 | 0.056 |
| Lysine | 0.76^a^ | 0.69^abc^ | 0.64^bcd^ | 0.72^ab^ | 0.58^cd^ | 0.54^d^ | 0.020 | <0.001 | <0.001 | 0.496 |
| Methionine | 0.20^a^ | 0.17^ab^ | 0.17^ab^ | 0.17^b^ | 0.17^ab^ | 0.17^b^ | 0.004 | 0.021 | 0.007 | 0.040 |
| Phenylalanine | 0.50^ab^ | 0.47^ab^ | 0.44^ab^ | 0.53^a^ | 0.42^ab^ | 0.41^b^ | 0.013 | 0.017 | 0.015 | 0.377 |
| Threonine | 0.55^a^ | 0.52^ab^ | 0.48^abc^ | 0.55^a^ | 0.46^bc^ | 0.45^c^ | 0.011 | 0.001 | <0.001 | 0.341 |
| Valine | 0.59 | 0.57 | 0.53 | 0.59 | 0.51 | 0.49 | 0.013 | 0.073 | 0.014 | 0.558 |
| ΣEAA | 4.80^a^ | 4.52^ab^ | 4.20^ab^ | 4.78^a^ | 3.98^b^ | 3.87^b^ | 0.102 | 0.003 | 0.001 | 0.440 |
| **NEAA** |  |  |  |  |  |  |  |  |  |  |
| Alanine | 0.59 | 0.58 | 0.52 | 0.58 | 0.51 | 0.50 | 0.011 | 0.061 | 0.009 | 0.719 |
| Aspartic acid | 1.11^a^ | 1.14^a^ | 1.02^ab^ | 1.10^ab^ | 0.97^ab^ | 0.94^b^ | 0.021 | 0.009 | 0.001 | 0.281 |
| Cysteine | 0.19 | 0.18 | 0.17 | 0.18 | 0.18 | 0.17 | 0.003 | 0.493 | 0.272 | 0.887 |
| Glutamic acid | 1.41^a^ | 1.34^ab^ | 1.37^a^ | 1.41^a^ | 1.17^bc^ | 1.16^c^ | 0.028 | <0.001 | <0.001 | 0.030 |
| Glycine | 0.67^a^ | 0.63^ab^ | 0.60^ab^ | 0.65^ab^ | 0.56^ab^ | 0.53^b^ | 0.016 | 0.020 | 0.002 | 0.400 |
| Proline | 0.58^a^ | 0.54^ab^ | 0.54^ab^ | 0.57^ab^ | 0.49^ab^ | 0.46^b^ | 0.013 | 0.041 | 0.005 | 0.298 |
| Serine | 0.51 | 0.51 | 0.46 | 0.51 | 0.44 | 0.43 | 0.011 | 0.052 | 0.011 | 0.490 |
| Tyrosine | 0.42 | 0.41 | 0.40 | 0.43 | 0.40 | 0.38 | 0.007 | 0.580 | 0.218 | 0.651 |
| ΣNEAA | 5.47^a^ | 5.31^ab^ | 5.08^abc^ | 5.43^a^ | 4.72^bc^ | 4.55^c^ | 0.097 | 0.003 | <0.001 | 0.178 |
| ΣAA | 10.27^a^ | 9.83^ab^ | 9.28^abc^ | 10.21^a^ | 8.70^bc^ | 8.42^c^ | 0.196 | 0.002 | <0.001 | 0.271 |
| ΣEAA/ΣNEAA | 0.38 | 0.37 | 0.35 | 0.38 | 0.36 | 0.36 | 0.003 | 0.055 | 0.108 | 0.738 |

FM = fish meal; BSFM = black soldier fly larvae meal; EAA = essential amino acid; NEAA = non-essential amino acid; AA = amino acid; SEM = standard error of the mean.

Within a row, means without a common superscript letter differ at *P* < 0.05, *n* = 4.

**Table S7** Fatty acid composition (mg/g) of hepatopancreas of mud crabs fed with different experimental diets for 8 weeks (wet basis).

| Fatty acids | Replacement of FM with BSFM, % | | | | | | SEM | *P*-value | | |
| --- | --- | --- | --- | --- | --- | --- | --- | --- | --- | --- |
|  | 0 | 10 | 20 | 30 | 40 | 60 |  | ANOVA | Linear | Quadratic |
| C12:00 | 3.14 | 3.41 | 3.22 | 3.35 | 3.10 | 2.97 | 0.074 | 0.610 | 0.312 | 0.225 |
| C14:00 | 4.73 | 4.64 | 4.33 | 4.60 | 4.19 | 4.20 | 0.095 | 0.427 | 0.076 | 0.943 |
| C16:00 | 17.27 | 17.37 | 17.12 | 17.68 | 16.95 | 16.94 | 0.161 | 0.823 | 0.530 | 0.541 |
| C18:00 | 5.76 | 6.08 | 5.60 | 6.21 | 6.07 | 6.04 | 0.082 | 0.284 | 0.242 | 0.835 |
| C20:00 | 0.34^d^ | 0.43^bcd^ | 0.40^cd^ | 0.47^abc^ | 0.53^ab^ | 0.56^a^ | 0.020 | <0.001 | <0.001 | 0.759 |
| ΣSFA | 31.25 | 31.92 | 30.68 | 32.30 | 30.83 | 30.72 | 0.328 | 0.666 | 0.555 | 0.542 |
| C16:1n | 5.46 | 5.47 | 5.32 | 5.10 | 5.29 | 5.73 | 0.087 | 0.470 | 0.759 | 0.099 |
| C18:1n-9 | 27.26^b^ | 28.18^ab^ | 29.29^ab^ | 29.84^ab^ | 28.88^ab^ | 29.55^ab^ | 0.287 | 0.046 | 0.010 | 0.087 |
| C20:1n-9 | 1.05 | 1.16 | 1.20 | 1.32 | 1.22 | 1.32 | 0.033 | 0.120 | 0.014 | 0.349 |
| C22:1n-11 | 0.35^d^ | 0.45^cd^ | 0.51^bc^ | 0.61^ab^ | 0.61^ab^ | 0.70^a^ | 0.029 | <0.001 | <0.001 | 0.178 |
| ΣMUFA | 34.11^b^ | 35.25^ab^ | 36.31^ab^ | 36.88^ab^ | 36.00^ab^ | 37.30^a^ | 0.358 | 0.039 | 0.010 | 0.318 |
| C18:2n-6 | 10.63 | 11.28 | 10.88 | 11.85 | 10.92 | 11.43 | 0.196 | 0.568 | 0.366 | 0.542 |
| C18:3n-6 | 0.26^c^ | 0.30^bc^ | 0.30^bc^ | 0.29^bc^ | 0.32^ab^ | 0.36^a^ | 0.008 | 0.001 | <0.001 | 0.212 |
| C20:2n-6 | 0.76 | 0.73 | 0.73 | 0.87 | 0.74 | 0.76 | 0.016 | 0.098 | 0.639 | 0.454 |
| C20:4n-6 | 0.83^bc^ | 1.04^bc^ | 0.80^c^ | 0.87^bc^ | 1.32^ab^ | 1.58^a^ | 0.078 | 0.001 | <0.001 | 0.008 |
| Σn-6PUFA | 12.48 | 13.35 | 12.71 | 13.88 | 13.29 | 14.13 | 0.223 | 0.237 | 0.051 | 0.992 |
| C18:3n-3 | 3.52 | 3.72 | 3.93 | 4.15 | 3.47 | 3.84 | 0.078 | 0.072 | 0.442 | 0.083 |
| C18:4n-3 | 0.48 | 0.53 | 0.51 | 0.52 | 0.47 | 0.47 | 0.017 | 0.872 | 0.516 | 0.410 |
| C20:4n-3 | 0.29 | 0.28 | 0.27 | 0.27 | 0.27 | 0.26 | 0.007 | 0.854 | 0.203 | 0.936 |
| C20:5n-3 | 9.03^ab^ | 9.37^a^ | 8.71^ab^ | 8.25^abc^ | 7.69^bc^ | 7.08^c^ | 0.220 | 0.003 | <0.001 | 0.167 |
| C22:5n-3 | 1.48 | 1.59 | 1.62 | 2.08 | 1.82 | 1.53 | 0.070 | 0.100 | 0.289 | 0.035 |
| C22:6n-3 | 7.16 | 7.44 | 7.37 | 7.43 | 7.01 | 6.98 | 0.078 | 0.314 | 0.180 | 0.103 |
| Σn-3PUFA | 21.97 | 22.95 | 22.41 | 22.69 | 20.72 | 20.15 | 0.336 | 0.055 | 0.015 | 0.044 |
| TFA | 99.81 | 103.47 | 102.11 | 105.75 | 100.83 | 102.29 | 0.974 | 0.640 | 0.705 | 0.292 |
| EPA + DHA | 16.19^ab^ | 16.82^a^ | 16.08^ab^ | 15.68^ab^ | 14.70^bc^ | 14.06^c^ | 0.275 | 0.010 | 0.001 | 0.107 |
| DHA/EPA | 0.34^b^ | 0.34^b^ | 0.36^b^ | 0.39^ab^ | 0.39^ab^ | 0.41^a^ | 0.007 | 0.002 | <0.001 | 0.556 |

FM = fish meal; BSFM = black soldier fly larvae meal; SFA=saturated fatty acids; MUFA = mono-unsaturated fatty acids; n-6PUFA = n-6 polyunsaturated fatty acids; n-3PUFA = n-3 polyunsaturated fatty acids; TFA = total fatty acids; EPA = eicosapentaenoic acid; DHA = docosahexaenoic acid; SEM = standard error of the mean.

Within a row, means without a common superscript letter differ at *P* < 0.05, *n* = 4.

**Table S8** Amino acid compositions (g/100 g) of muscle of mud crabs fed with different experimental diets for 8 weeks (wet basis).

| Amino acids | Replacement of FM with BSFM, % | | | | | | SEM | *P*-value | | |
| --- | --- | --- | --- | --- | --- | --- | --- | --- | --- | --- |
|  | 0 | 10 | 20 | 30 | 40 | 60 |  | ANOVA | Linear | Quadratic |
| **EAA** |  |  |  |  |  |  |  |  |  |  |
| Arginine | 1.99 | 1.98 | 1.97 | 2.00 | 1.95 | 1.93 | 0.013 | 0.718 | 0.244 | 0.454 |
| Histidine | 0.40 | 0.36 | 0.39 | 0.39 | 0.38 | 0.38 | 0.004 | 0.127 | 0.760 | 0.477 |
| Isoleucine | 0.74 | 0.70 | 0.70 | 0.72 | 0.69 | 0.71 | 0.007 | 0.554 | 0.251 | 0.301 |
| Leucine | 1.35 | 1.31 | 1.34 | 1.34 | 1.29 | 1.32 | 0.011 | 0.766 | 0.471 | 0.924 |
| Lysine | 1.42 | 1.40 | 1.42 | 1.43 | 1.35 | 1.37 | 0.012 | 0.344 | 0.102 | 0.573 |
| Methionine | 0.25^b^ | 0.27^b^ | 0.35^a^ | 0.37^a^ | 0.36^a^ | 0.40^a^ | 0.015 | <0.001 | <0.001 | 0.092 |
| Phenylalanine | 0.72 | 0.70 | 0.71 | 0.71 | 0.69 | 0.71 | 0.004 | 0.471 | 0.312 | 0.354 |
| Threonine | 0.76 | 0.74 | 0.76 | 0.76 | 0.72 | 0.73 | 0.006 | 0.450 | 0.204 | 0.527 |
| Valine | 0.79 | 0.77 | 0.80 | 0.79 | 0.76 | 0.77 | 0.007 | 0.326 | 0.417 | 0.553 |
| ΣEAA | 8.43 | 8.22 | 8.50 | 8.51 | 8.18 | 8.30 | 0.068 | 0.639 | 0.652 | 0.620 |
| **NEAA** |  |  |  |  |  |  |  |  |  |  |
| Alanine | 1.16 | 1.12 | 1.12 | 1.13 | 1.09 | 1.12 | 0.009 | 0.476 | 0.231 | 0.285 |
| Aspartic acid | 1.76 | 1.69 | 1.78 | 1.75 | 1.69 | 1.70 | 0.015 | 0.297 | 0.298 | 0.518 |
| Cysteine | 0.13^b^ | 0.14^ab^ | 0.15^ab^ | 0.15^ab^ | 0.14^ab^ | 0.16^a^ | 0.003 | 0.004 | 0.001 | 0.065 |
| Glutamic acid | 3.00^a^ | 2.83^b^ | 2.93^ab^ | 2.85^b^ | 2.81^ab^ | 2.79^b^ | 0.024 | 0.031 | 0.009 | 0.663 |
| Glycine | 1.49 | 1.54 | 1.46 | 1.52 | 1.41 | 1.40 | 0.019 | 0.153 | 0.038 | 0.318 |
| Proline | 1.00 | 1.02 | 1.02 | 1.03 | 1.00 | 1.04 | 0.008 | 0.718 | 0.583 | 0.762 |
| Serine | 0.68 | 0.67 | 0.69 | 0.68 | 0.65 | 0.66 | 0.005 | 0.446 | 0.267 | 0.547 |
| Tyrosine | 0.79 | 0.77 | 0.80 | 0.79 | 0.76 | 0.77 | 0.007 | 0.326 | 0.417 | 0.553 |
| ΣNEAA | 10.01 | 10.03 | 10.05 | 10.03 | 9.50 | 9.65 | 0.076 | 0.097 | 0.022 | 0.302 |
| ΣAA | 18.44 | 18.25 | 18.55 | 18.54 | 17.68 | 17.96 | 0.136 | 0.366 | 0.158 | 0.438 |
| ΣEAA/ΣNEAA | 0.22^ab^ | 0.21^b^ | 0.23^a^ | 0.23^a^ | 0.22^ab^ | 0.23^a^ | 0.002 | 0.012 | 0.004 | 1.000 |

FM = fish meal; BSFM = black soldier fly larvae meal; EAA = essential amino acid; NEAA = non-essential amino acid; AA = amino acid; SEM = standard error of the mean.

Within a row, means without a common superscript letter differ at *P* < 0.05, *n* = 4.

**Table S9** Fatty acid composition (mg/g) of muscle of mud crabs fed with different experimental diets for 8 weeks (wet basis).

| Fatty acids | Replacement of FM with BSFM, % | | | | | | SEM | *P*-value | | |
| --- | --- | --- | --- | --- | --- | --- | --- | --- | --- | --- |
|  | 0 | 10 | 20 | 30 | 40 | 60 |  | ANOVA | Linear | Quadratic |
| C12:00 | 0.01 | 0.01 | 0.01 | 0.01 | 0.01 | 0.01 | 0.000 | 0.366 | 0.142 | 0.504 |
| C14:00 | 0.05 | 0.05 | 0.05 | 0.05 | 0.04 | 0.05 | 0.002 | 0.973 | 0.456 | 0.984 |
| C16:00 | 1.08 | 1.11 | 1.16 | 1.09 | 1.01 | 1.12 | 0.023 | 0.642 | 0.737 | 0.809 |
| C18:00 | 0.68 | 0.62 | 0.71 | 0.63 | 0.65 | 0.65 | 0.017 | 0.764 | 0.765 | 0.948 |
| C20:00 | 0.02 | 0.02 | 0.02 | 0.02 | 0.02 | 0.02 | 0.000 | 0.691 | 0.293 | 0.728 |
| ΣSFA | 1.83 | 1.80 | 1.94 | 1.80 | 1.73 | 1.84 | 0.038 | 0.793 | 0.708 | 0.874 |
| C16:1n | 0.12 | 0.11 | 0.11 | 0.12 | 0.11 | 0.11 | 0.002 | 0.111 | 0.084 | 0.115 |
| C18:1n-9 | 1.19 | 1.18 | 1.20 | 1.23 | 1.12 | 1.17 | 0.020 | 0.804 | 0.544 | 0.761 |
| C20:1n-9 | 0.04 | 0.04 | 0.04 | 0.04 | 0.04 | 0.04 | 0.001 | 0.368 | 0.388 | 0.908 |
| C22:1n-11 | 0.02 | 0.02 | 0.02 | 0.02 | 0.02 | 0.02 | 0.001 | 0.110 | 0.213 | 0.709 |
| ΣMUFA | 1.37 | 1.36 | 1.37 | 1.41 | 1.28 | 1.35 | 0.021 | 0.686 | 0.537 | 0.833 |
| C18:2n-6 | 0.44 | 0.39 | 0.46 | 0.45 | 0.41 | 0.43 | 0.017 | 0.898 | 0.942 | 0.761 |
| C18:3n-6 | 0.02 | 0.01 | 0.02 | 0.02 | 0.02 | 0.02 | 0.000 | 0.610 | 0.264 | 0.819 |
| C20:2n-6 | 0.07 | 0.06 | 0.07 | 0.07 | 0.06 | 0.07 | 0.002 | 0.921 | 0.890 | 0.793 |
| C20:4n-6 | 0.26 | 0.24 | 0.26 | 0.24 | 0.26 | 0.33 | 0.010 | 0.104 | 0.044 | 0.045 |
| Σn-6PUFA | 0.78 | 0.72 | 0.81 | 0.78 | 0.76 | 0.84 | 0.019 | 0.563 | 0.316 | 0.537 |
| C18:3n-3 | 0.07 | 0.08 | 0.09 | 0.08 | 0.07 | 0.08 | 0.003 | 0.628 | 0.611 | 0.453 |
| C18:4n-3 | 0.01 | 0.01 | 0.01 | 0.01 | 0.01 | 0.01 | 0.000 | 0.981 | 0.704 | 0.914 |
| C20:4n-3 | 0.02 | 0.01 | 0.01 | 0.01 | 0.01 | 0.01 | 0.001 | 0.052 | 0.004 | 0.138 |
| C20:5n-3 | 1.50 | 1.52 | 1.61 | 1.39 | 1.38 | 1.40 | 0.037 | 0.443 | 0.166 | 0.657 |
| C22:5n-3 | 0.07^b^ | 0.11^ab^ | 0.12^a^ | 0.11^a^ | 0.11^a^ | 0.13^a^ | 0.005 | 0.008 | 0.001 | 0.064 |
| C22:6n-3 | 0.83 | 0.83 | 0.95 | 0.82 | 0.80 | 0.85 | 0.029 | 0.796 | 0.821 | 0.675 |
| Σn-3PUFA | 2.50 | 2.55 | 2.78 | 2.41 | 2.39 | 2.47 | 0.065 | 0.609 | 0.487 | 0.582 |
| TFA | 6.48 | 6.42 | 6.91 | 6.39 | 6.15 | 6.51 | 0.127 | 0.746 | 0.679 | 0.790 |
| EPA + DHA | 2.33 | 2.35 | 2.56 | 2.20 | 2.18 | 2.25 | 0.063 | 0.606 | 0.362 | 0.649 |
| DHA/EPA | 0.14 | 0.15 | 0.16 | 0.16 | 0.15 | 0.16 | 0.003 | 0.732 | 0.268 | 0.470 |

FM = fish meal; BSFM = black soldier fly larvae meal; SFA=saturated fatty acids; MUFA = mono-unsaturated fatty acids; n-6PUFA = n-6 polyunsaturated fatty acids; n-3PUFA = n-3 polyunsaturated fatty acids; TFA = total fatty acids; EPA = eicosapentaenoic acid; DHA = docosahexaenoic acid; SEM = standard error of the mean.

Within a row, means without a common superscript letter differ at *P* < 0.05, *n* = 4.

**Table S10** Flavor nucleotides contents (mg/100 g) in the muscle of mud crabs fed with different experimental diets for 8 weeks (wet basis)^1^.

| Flavor nucleotides | Taste | Replacement of FM with BSFM, % | | | | | | SEM | *P*-value | | |
| --- | --- | --- | --- | --- | --- | --- | --- | --- | --- | --- | --- |
|  |  | 0 | 10 | 20 | 30 | 40 | 60 |  | ANOVA | Linear | Quadratic |
| AMP | Umami/sweet (+) | 83.98 | 89.17 | 71.91 | 62.39 | 67.02 | 70.05 | 3.373 | 0.145 | 0.033 | 0.270 |
| GMP | Umami (+) | 5.31^bc^ | 5.68^abc^ | 6.14^ab^ | 6.25^a^ | 5.48^abc^ | 5.15^c^ | 0.119 | 0.009 | 0.437 | 0.001 |
| IMP | Umami (+) | 254.74 | 252.80 | 259.54 | 269.39 | 254.99 | 236.92 | 4.302 | 0.453 | 0.425 | 0.112 |

FM = fish meal; BSFM = black soldier fly larvae meal; AMP = adenosine monophosphate; GMP = guanosine monophosphate; IMP = inosine monophosphate; SEM = standard error of the mean.

Within a row, means without a common superscript letter differ at *P* < 0.05, *n* = 4.

^1^The type of nucleotide and associated flavor refer to Guo et al., (2014).

**Table S11** Eigen analysis of the covariance matrix loadings for significant principal components.

| Items | Factor 1 | Factor 2 | Factor 3 |
| --- | --- | --- | --- |
| UFAA | 0.33 | 0.21 | –0.11 |
| SFAA | –0.19 | 0.33 | 0.28 |
| BFAA | 0.16 | 0.40 | 0.25 |
| AMP | 0.29 | –0.14 | 0.51 |
| GMP | –0.04 | 0.45 | 0.07 |
| IMP | 0.04 | 0.31 | –0.58 |
| Hypoxanthine | –0.23 | 0.22 | 0.41 |
| Sourness | –0.39 | 0.06 | –0.09 |
| Bitterness | –0.40 | –0.04 | 0.09 |
| Sweetness | 0.26 | –0.30 | –0.11 |
| Umami | 0.31 | 0.34 | –0.06 |
| Richness | 0.30 | –0.25 | 0.20 |
| Saltiness | 0.36 | 0.23 | 0.03 |
| Percentage of variance, % | 38.09 | 23.13 | 9.98 |
| Cumulative, % | 38.09 | 61.22 | 71.20 |

UFAA = umami free amino acids; SFAA = sweet free amino acids; BFAA = bitter free amino acid; AMP = adenosine monophosphate; GMP = guanosine monophosphate; IMP = inosine monophosphate.
